# Whole Transcriptome Analysis of Renal Intercalated Cells Predicts Lipopolysaccharide Mediated Inhibition of Retinoid X Receptor alpha Function

**DOI:** 10.1038/s41598-018-36921-z

**Published:** 2019-01-24

**Authors:** Vijay Saxena, James Fitch, John Ketz, Peter White, Amy Wetzel, Melinda A. Chanley, John D. Spencer, Brian Becknell, Keith R. Pierce, Sam W. Arregui, Raoul D. Nelson, George J. Schwartz, Victoria Velazquez, Logan A. Walker, Xi Chen, Pearlly Yan, David S. Hains, Andrew L. Schwaderer

**Affiliations:** 1 Indiana University School of Medicine, Riley Children’s Hospital, Indianapolis, Indiana United States; 2 The Institute for Genomic Medicine, Nationwide Children’s Hospital, Columbus, Ohio United States; 3 The Research Institute at Nationwide Children’s, Center for Clinical and Translational Research, Columbus, Ohio, and College of Medicine, Ohio State University, Columbus, Ohio United States; 4 Department of Pediatrics, College of Medicine, The Ohio State University, Columbus, Ohio United States; 5 Innate Immunity Translational Research Center, Children’s Foundation Research Institute at Le Bonheur Children’s Hospital, Memphis, Tennessee United States; 6 Division of Nephrology, Department of Pediatrics, University of Utah, Salt Lake City, Utah United States; 7 University of Rochester Medical Center, School of Medicine and Dentistry, Rochester, New York United States; 8 Research Institute at Nationwide Children’s Hospital Flow Cytometry Core Laboratory, Columbus, Ohio, United States; 9 Department of Physics, College of Arts and Sciences, The Ohio State University, Columbus, Ohio, United States; 10 Genomics Shared Resource, The Ohio State University Comprehensive Cancer Center, Columbus, Ohio, United States; 11 Division of Hematology, Department of Internal Medicine, The Ohio State University Wexner Medical Center, Columbus, Ohio, United States

## Abstract

The renal collecting duct consists of intercalated cells (ICs) and principal cells (PCs). We have previously demonstrated that collecting ducts have a role in the innate immune defense of the kidney. Transcriptomics is an important tool used to enhance systems-level understanding of cell biology. However, transcriptomics performed on whole kidneys provides limited insight of collecting duct cell gene expression, because these cells comprise a small fraction of total kidney cells. Recently we generated reporter mouse models to enrich collecting duct specific PC and ICs and reported targeted gene expression of anti-microbial peptide genes. Here we report transcriptomics on enriched ICs and PCs and performed a pilot study sequencing four single ICs. We identified 3,645 genes with increased relative expression in ICs compared to non-ICs. In comparison to non-PCs, 2,088 genes had higher relative expression in PCs. IC associated genes included the innate interleukin 1 receptor, type 1 and the antimicrobial peptide(AMP) adrenomedullin. The top predicted canonical pathway for enriched ICs was lipopolysaccharide/Interleukin 1 mediated inhibition of Retinoid X Receptor alpha function and decreased Retinoid X Receptor expression was confirmed to occur 1-hour post experimental murine UTI in ICs but not in non-ICs.

## Introduction

The renal collecting duct contains intercalated cells (ICs) and principal cells (PCs). PCs express aquaporin 2 (AQP2),modulate water and electrolyte reabsorption while ICs express the B1 subunit of vacuolar H^+^-ATPase (V-ATPase-B1) and maintain acid-base homeostasis^1,2^. We, and others, have demonstrated that ICs are involved in the renal bacterial defense^3–7^. The expansion of collecting duct function to include innate immunity indicates that IC and/or PC functions are more diverse than previously recognized.

Transcriptomics is a critical component of systems-level understanding of cell biology^8^. However, analysis of collecting duct cell function by transcriptomics performed on whole kidneys or tubular segments is limited because the kidney is a conglomerate of many cell types. For instance, the kidney contains cells from the proximal tubule (PTCs), loop of Henle (LOH), distal convoluted tubule, connecting tubule, collecting duct, glomerulus along with vascular, interstitial and resident immune cells. Further, we had previously reported that ICs account for ~1% of cortical cells and ~2% of medullary cells while PCs accounted for ~2% of cortical cells ~20% of medullary cells in the murine kidney^3^. Therefore, IC and/or PC expression risks being diluted by numerous cell types. Past techniques to enrich collecting duct cells have included dissecting the relatively collecting-duct rich medulla from the collecting duct poor cortex or, pooling microdissected tubules from different nephron segments or use of cell cultures^9–11^. While the collecting duct can be evaluated using these aforementioned methodologies, the distinct cell type (e.g. ICs and PCs) cannot be analyzed individually by dissecting out the medulla or tubular segments and cultured cells may not retain the phenotype of the targeted cell type.

Generation of transgenic mice expressing cre recombinase under the control of IC and PC specific promoters provides a modality for the evaluation of collecting duct innate immunity at the cellular level^12,13^. We have reported on methodologies using two fluorescent reporter mice, V-ATPase B1-cre^+^tdTomato^+^ mice to label ICs and AQP2-cre^+^tdTomato^+^ mice to label PCs and then flow sorting to enrich viable tdTomato^+^ PCs and ICs for analysis^14^. The objective of this study is to identify distinct and overlapping transcriptome profiles associated with ICs and PCs.

## Results

### Quality control

To determine if there were any unexpected issues and to help ensure that the observed differences in expression were due to experimental conditions, a principal component analysis plot (PCA), volcano plot and log intensity ratio (M-value or MA) plot were generated for the groups: IC versus non-ICs (IC vs non-IC), PC versus non-PCs (PC vs non-PC) and ICs versus PCs (Supplemental Data S1). The PCA plots showed that the samples segregate by sample group, indicating that expression levels were not affected by something other than the intended treatment (like a batch effect). The volcano plots for global gene expression demonstrated a large number of statistically significant differentially expressed genes with more genes with lower relative expression ICs or PCs compared to non-ICs and non-PCs. Additionally, characteristic MA plot shapes were noted.

### Relative enrichment of IC and PC cells

**Figure 1:**
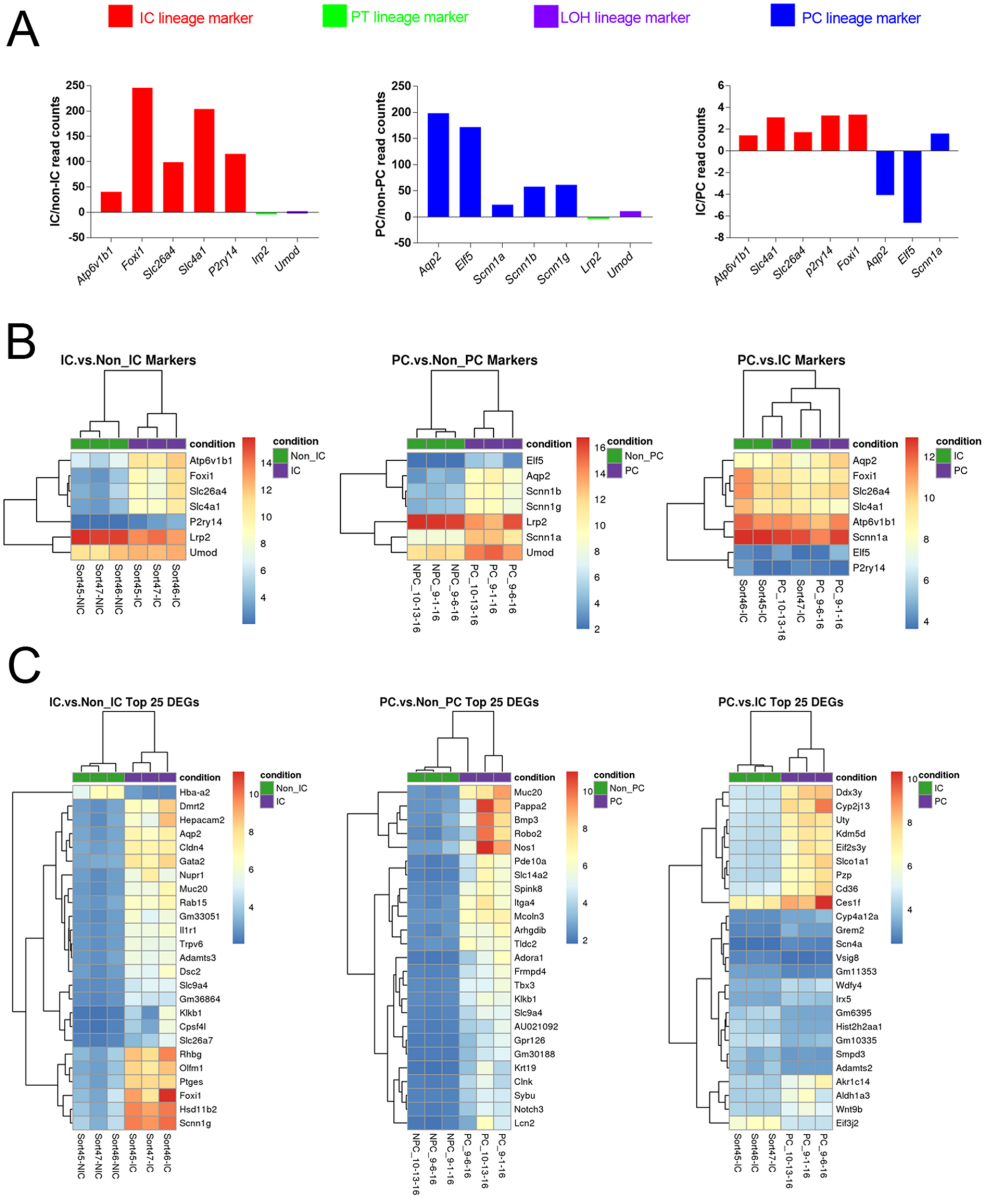
(**A**) Relative lineage marker fold change. IC lineage markers (left panel) mRNA expression was increased 41 to 246-fold in ICs compared to non-ICs, while PC lineage marker mRNA expression (middle panel) was increased 24 to198-fold in PCs compared to non-PCs (middle panel). Of note, *Scnn1a* did not appear to be as enriched in PCs compared to other PC markers. In the direct IC to PC comparison (right panel) IC markers were around ≥ 1.5-fold higher in ICs than PCs. PC markers were around ≥4-fold higher in PCs compared to ICs with the exception of *Scnn1a* which was higher in ICs. (**B**) Heatmaps of the lineage markers are presented IC vs NICs (left panel), PC vs non-PCs (middle panel) and ICs vs PCs (right panel) showing relative expression. (**C**) Heatmaps of the 25 most differentially expressed genes in ICs vs non-ICs (left panel), PCs vs non-PCs (middle panel) and ICs vs PCs (right panel are presented). The samples are grouped by dendrogram at the top of figure and genes are grouped by the dendrogram on the left side of the figure. Similarly regulated genes are grouped together. The color coded legend bar indicate normalized read counts that have been log2 transformed. Red indicates higher expression while blue indicate lower expression.

IC and PC cells were enriched from IC and PC reporter mice by enzymatic digestion of kidney followed by flow sorting of Tdtomato positive (presumed ICs and PCs) and negative (presumed non-ICs and non-PCs) cells then RNA-Seq was carried out to determine relative abundance for all expressed genes in IC and PC compared to non-ICs and non-PCs. Lineage markers known to be widely expressed by these cells were used to determine the degree IC and PCs were enriched. IC lineage markers included the following genes (corresponding proteins in parenthesis): *Atp6v1b1* (V-ATPase B1), *Slc4a1* (Anion exchanger 1), *Slc26a4* (pendrin), *P2ry14 (*P2Y purinoceptor 14) and *Foxi1 (*forkhead box i1)^15–17^. PC lineage markers included *Aqp2*, *Elf2* (E74-like factor 2), *Scnn1a*, *Scnn1b* and *Scnn1g* (alpha, beta and gamma subunits respectively for the epithelial sodium channel, ENaC)^18,19^. *Lrp2* (megalin) was used as a proximal tubule marker and *Umod* (Tamm-Horsfall protein) was used as a loop of Henle marker^20,21^. The RNA-Seq groups lineage marker comparison results are presented in as IC/non-IC and PC/non-PC read counts in Fig. 1A and similar markers are presented as differentially expressed genes as heatmap in Fig. 1B.

### IC versus non-ICs

#### Genes enriched in ICs

Compared to non-ICs, 3,645 genes had higher relative expression by ≥2-fold with a p-value < 0.05 in ICs. The top 25 genes (excluding long non-coding genes) with highest fold change increase in ICs are presented in Table 1 and a heatmap of the top 25 differentially expressed genes is presented in Fig. 1C (left panel). The complete list of genes is presented at https://hpcwebapps.cit.nih.gov/ESBL/Database/IC/index.html. The top genes with increased relative expression in ICs included those previously reported to be relatively enriched in ICs compared to PCs including doublesex and mab-3 related transcription factor 2 (*Dmrt2*) and *Foxi1*, but also traditional principal markers such as *Aqp2*^22^.

**Table 1:** Top 25 gene with highest relative expression in ICs versus non-ICs.

Gene Symbol	Gene Description	Fold Change	p-Adjusted
*Dmrt2*	doublesex and mab-3 related transcription factor 2	1402.61	<0.001
*Muc20*	mucin 20	638.64	<0.001
*Aqp2*	aquaporin 2	497.92	<0.001
*Il1r1*	interleukin 1 receptor, type I	481.87	<0.001
*Trpv6*	transient receptor potential cation channel, subfamily V, member 6	446.16	<0.001
*Olfm1*	olfactomedin 1	423.47	<0.001
*Rab15*	RAB15, member RAS oncogene family	397.74	<0.001
*Nupr1*	nuclear protein transcription regulator 1	388.64	<0.001
*Hepacam2*	HEPACAM family member 2	381.31	<0.001
*Rhbg*	Rhesus blood group-associated B glycoprotein	367.17	<0.001
*Cldn4*	claudin 4	345.36	<0.001
*Klkb1*	kallikrein B, plasma 1	321.30	0.022
*Gata2*	GATA binding protein 2	317.62	<0.001
*Slc9a4*	solute carrier family 9 (sodium/hydrogen exchanger), member 4	311.26	<0.001
*Cpsf4l*	cleavage and polyadenylation specific factor 4-like	289.04	0.003
*Hsd11b2*	hydroxysteroid 11-beta dehydrogenase 2	275.03	<0.001
*Foxi1*	forkhead box i1	256.91	0.003
*Scnn1g*	sodium channel, non- voltage-gated 1 gamma	246.28	<0.001
*Ptges*	Prostaglandin E synthase	234.42	<0.001
*Dsc2*	desmocollin 2	228.71	<0.001
*Slc26a7*	solute carrier family 26, member 7	221.91	<0.001
*Adamts3*	a disintegrin-like and metallopeptidase (reprolysin type) with thrombospondin type 1 motif, 3	216.11	<0.001
*Fgf12*	fibroblast growth factor 12	213.06	<0.001
*Rasd2*	RASD family, member 2	205.90	<0.001
*Kcns3*	potassium voltage-gated channel, delayed-rectifier, subfamily S, member 3	196.7	<0.001

#### Canonical pathways

**Figure 2:**
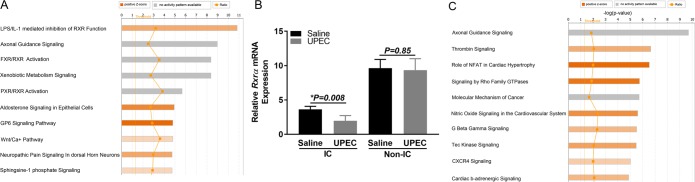
(**A**) The top 10 canonical pathways associated with IC mRNA expression are presented. (**B**) IC specific inhibition of *Rxrα* mTNA expression. IC reporter mice were infected with UPEC or saline and Tdt^+^ (IC) and Tdt^−^ (Non-IC) cells were enriched. *Rxrα* mRNA was measured normalized to GAPDH. IC cells showed significant inhibition of mRNA expression upon UPEC exposure (3.64 ± 0.44 in saline vs. 1.97 ± 0.85 in UPEC, *P* = *0.008*) compared to NIC (9.63 ± 1.26 in saline vs. 9.34 ± 1.67 in UPEC, *P* = *0.85*) cells. N = 4 experiments, each using a separate sort consisting of kidneys from 1–2 mice. (**C)** The top 10 canonical pathways associated with PC mRNA expression are presented.

Ingenuity analysis predicted key pathways associated with ICs. The top 10 canonical pathways are presented in Fig. 2A. The top predicted pathway in ICs was “interaction between lipopolysaccharide (LPS)/interleukin -1 (IL-1) mediated inhibition of Retinoid X receptor (RXR) function”. Key IC associated innate immune genes involved in this pathway are presented in Supplemental Data S2. Other involved pathways included wingless-type MMTV integration site family member (Wnt)/Ca^++^, axonal guidance signaling and sphingosine-1 phosphate signaling.

### Functional evaluation of LPS/IL-1 mediated inhibition of RXR function in IC versus non-ICs

Retinoid X Receptors have been implicated to play key role in regulating variety of cell function such as cell differentiation, immune response, and metabolism^23^. One of its member, RXR which is a key nuclear receptor in metabolic processes has been shown to be down regulated during viral infections and protect host^24^. Ingenuity analysis predicted top pathway (inhibition of RXR function) in ICs was functionally validated in murine model of UTI. When ICs were enriched from IC reporter mice 1 hr post transurethral inoculation of UPEC vs. saline control, ICs but not non-ICs significantly down regulated *Rxrα* mRNA expression with UPEC vs. saline (Fig. 2B). Our findings indicate that ICs may use this mechanism to protect the host from transurethral infections.

### PC versus non-PCs

#### Genes enriched in PCs

Compared to non-PCs, 2088 genes had higher relative expression in PCs by ≥2-fold and an adjusted p value < 0.05. The 25 genes with highest fold change increase in PCs are presented in Table 2. A heatmap of the 25 most differentially expressed genes is presented in Fig. 1B (middle panel). The complete list of genes is presented at https://hpcwebapps.cit.nih.gov/ESBL/Database/PC/index.html. Genes with higher expression in PCs compared to non-PCs included mucin 20 (*Muc20*), urea transporters including solute carrier family 14 member 2 (*Slc14a2*) and renal development genes such as bone morphogenetic protein 3 (*Bmp3*), and neurogenic locus notch homolog protein 3 (*Notch 3*).

**Table 2:** Top 25 genes with highest relative expression in PCs versus non-PCs.

Gene Symbol	Gene Description	Fold Change	p-Adjusted
*Muc20*	mucin 20	2739.04	<0.001
*Pappa2*	pappalysin 2	1294.57	<0.001
*Spink8*	serine peptidase inhibitor, Kazal type 8	723.69	<0.001
*Pde10a*	phosphodiesterase 10A	694.92	<0.001
*Robo2*	roundabout homolog 2 (Drosophila)	675.41	0.021
*Slc14a2*	solute carrier family 14 (urea transporter), member 2	667.44	<0.001
*Nos1*	nitric oxide synthase 1, neuronal	655.19	<0.001
*Bmp3*	bone morphogenetic protein 3	546.92	0.011
*Itga4*	integrin alpha 4	545.16	<0.001
*Mcoln3*	mucolipin 3	533.56	<0.001
*Adora1*	adenosine A1 receptor	525.07	<0.001
*Klkb1*	kallikrein B, plasma 1	409.54	<0.001
*Frmpd4*	FERM and PDZ domain containing 4	403.22	<0.001
*Tbx3*	T-box 3	396.27	<0.001
*Arhgdib*	Rho, GDP dissociation inhibitor (GDI) beta	362.66	<0.001
*Tldc2*	TBC/LysM associated domain containing 2	326.32	<0.001
*Slc9a4*	solute carrier family 9 (sodium/hydrogen exchanger), member 4	326.09	<0.001
*Krt19*	keratin 19	313.32	<0.001
*AU021092*	expressed sequence AU021092	271.27	<0.001
*Clnk*	cytokine-dependent hematopoietic cell linker	269.25	<0.001
*Gpr126*	G protein-coupled receptor 126	259.86	<0.001
*Lcn2*	lipocalin 2	249.00	<0.001
*Sybu*	syntabulin (syntaxin-interacting)	246.59	<0.001
*Notch3*	notch 3	241.99	<0.001
*Prkar1b*	protein kinase, cAMP dependent regulatory, type I beta	219.15	

#### Canonical pathways

Ingenuity software identified key pathways associated with PCs. The top 10 canonical pathways enriched in PCs are presented in Fig. 2C. The top pathway included axonal guidance signaling among others as thrombin signaling and CXCR4 signaling.

### IC versus PC

In direct IC to PC comparison 123 genes were differentially expressed (adjusted p < 0.05 and fold-change ≥2). The complete list of genes is presented at https://hpcwebapps.cit.nih.gov/ESBL/Database/PC/PC-IC%20Results.html. ICs and PCs appeared more similar to each other than non-ICs or non-PCs respectively (Fig. 1B,C).

#### Differentially expressed genes in IC

Twenty genes with higher fold change in ICs vs. PCs are listed in Table 3 excluding predicted genes. A heatmap of top 25 differentially expressed genes in ICs vs. PCs is presented in Fig. 1C (right panel).

**Table 3:** Top 20 genes overexpressed in ICs compared to PCs.

Gene Symbol	Gene Description	Fold Change	p-Adjusted
*Smpd3*	sphingomyelin phosphodiesterase 3, neutral	25.09	0.016
*Hist2h2aa1*	histone cluster 2, H2aa1	17.56	<0.001
*Eif3j2*	eukaryotic translation initiation factor 3, subunit J2	15.20	<0.001
*Adamts2*	a disintegrin-like and metallopeptidase (reprolysin type) with thrombospondin type 1 motif, 2	13.65	0.031
*Dapl1*	death associated protein-like 1	8.70	0.043
*Rn7s2*	7S RNA 2	8.32	<0.001
*Rpl23a-ps3*	ribosomal protein L23A, pseudogene 3	7.71	0.040
*Rpl19-ps11*	ribosomal protein L19, pseudogene 11	6.34	<0.001
*Tpm3-rs7*	tropomyosin 3, related sequence 7	5.33	<0.001
*Nsa2-ps1*	NSA2 ribosome biogenesis homolog, pseudogene 1	5.18	<0.001
*Rasd1*	RAS, dexamethasone-induced 1	4.27	<0.001
*Adm*	adrenomedullin	4.01	0.016
*Erdr1*	erythroid differentiation regulator 1	3.91	0.035
*Oaz1-ps*	ornithine decarboxylase antizyme 1, pseudogene	3.60	<0.001
*Ryr2*	ryanodine receptor 2, cardiac	3.20	0.006
*Tbx2*	T-box 2	2.48	<0.001
*A4galt*	alpha 1,4-galactosyltransferase	2.40	0.003
*Tbc1d1*	TBC1 domain family, member 1	2.16	0.001
*Gsta4*	glutathione S-transferase, alpha 4	2.06	0.005
*Anapc13*	anaphase promoting complex subunit 13	1.91	0.001

#### Differentially expressed genes in PC

Genes with higher relative expression in PCs compared to ICs ranked by fold change included water channels such as *Aqp4* (10.6-fold higher in PCs, p = 0.0006) along with proteins involved in tissue development, regeneration and cell polarity such as *Wnt9b (*16.5 fold, p < 0.0001. The top 20 genes with higher expression in PCs compared to ICs is presented in Table 4.

**Table 4:** Top 20 genes overexpressed in PCs compared to ICs.

Gene Symbol	Gene Description	Fold Change	p-Adjusted
*Ddx3y*	DEAD (Asp-Glu-Ala-Asp) box polypeptide 3, Y-linked	1916.66	<0.001
*Cyp2j13*	cytochrome P450, family 2, subfamily j, polypeptide 13	1537.46	<0.001
*Uty*	ubiquitously transcribed tetratricopeptide repeat gene, Y chromosome	1448.34	<0.001
*Slco1a1*	solute carrier organic anion transporter family, member 1a1	1409.07	<0.001
*Kdm5d*	lysine (K)-specific demethylase 5D	1158.52	<0.001
*Eif2s3y*	eukaryotic translation initiation factor 2, subunit 3, structural gene Y-linked	1141.56	<0.001
*Pzp*	pregnancy zone protein	1141.01	<0.001
*Akr1c14*	aldo-keto reductase family 1, member C14	75.36	<0.001
*Cyp4a12a*	cytochrome P450, family 4, subfamily a, polypeptide 12a	51.90	0.013
*Grem2*	gremlin 2 homolog, cysteine knot superfamily (Xenopus laevis)	47.25	0.040
*Wdfy4*	WD repeat and FYVE domain containing 4	36.63	<0.001
*Cd36*	CD36 antigen	36.32	<0.001
*Aldh1a3*	aldehyde dehydrogenase family 1, subfamily A3	19.65	<0.001
*Wnt9b*	wingless-type MMTV integration site family, member 9B	16.46	<0.001
*Ces1f*	carboxylesterase 1F	15.66	<0.001
*Enox1*	ecto-NOX disulfide-thiol exchanger 1	11.51	0.040
*Aqp4*	aquaporin 4	10.60	<0.001
*Itga4*	integrin alpha 4	10.49	0.001
*Timd2*	T cell immunoglobulin and mucin domain containing 2	9.12	0.033
*Ugt2b38*	UDP glucuronosyltransferase 2 family, polypeptide B38	8.62	0.040

#### Innate immune genes

Innate immune genes were identified by keyword search such as“Toll like receptor” (*Tlr*), “nod like receptor” (*Nlr*), “interleukin receptor” (*Ilr*), “cluster of differentiation receptor” (*Cd*), “chemokine receptor”, “defensin”, “ribonuclease”, and “calcium binding proteins” from unfiltered comprehensive gene list (Supplemental Data S3). Most innate immune genes did not have significantly different relative expression between ICs and PCs when using adjusted p-values. Secretogranin 5 (*Scg5*), an antimicrobial peptide, had higher relative expression in ICs by RT-PCR validation (Supplemental Data S4).

### *In situ* and *immunofluorescent* evaluation of collecting duct marker expression and evaluation of cre lines

**Figure 3:**
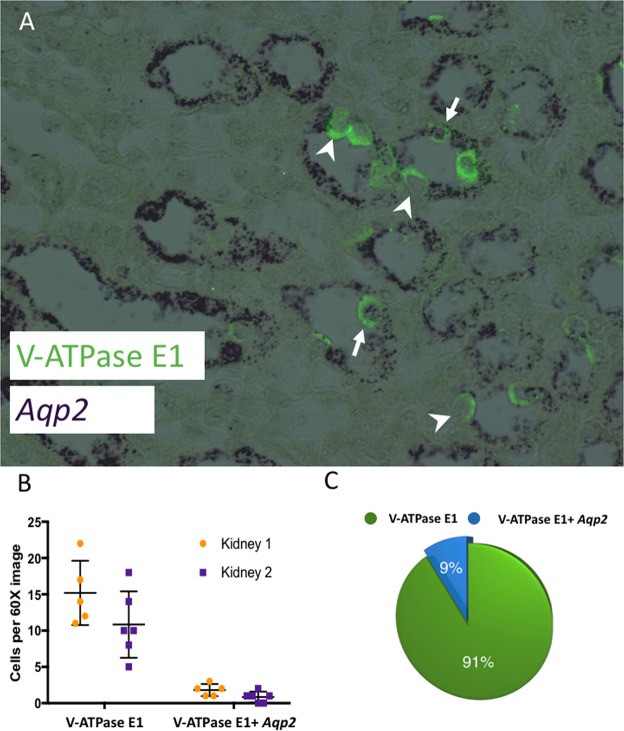
(**A**) Co-expression of *Aqp2* mRNA and V-ATPase E1 protein in wild type C57Bl/6 mice. Imaging of mouse kidney sections for *Aqp2* mRNA *in-situ* hybridization (purple) and overlaid with immunofluorescent protein labeling of V-ATPase E1 (green). The majority of ICs did not co-label for *Aqp2* mRNA (arrowheads) but a subset did (arrows), Magnification 60X. (**B**) Kidneys from 2 mice were evaluated and the cellular expression of V-ATPase E1 alone and both V-ATPase E1 and *Aqp2* expression was found to be similar between kidneys, p values 0.08 and 0.86 respectively. The horizontal bar represents the mean and the error bars represent the standard deviation. (**C**) Approximately 9% of cells with V-ATPase E1 protein expression also had expression for *Aqp2* mRNA.

**Figure 4:**
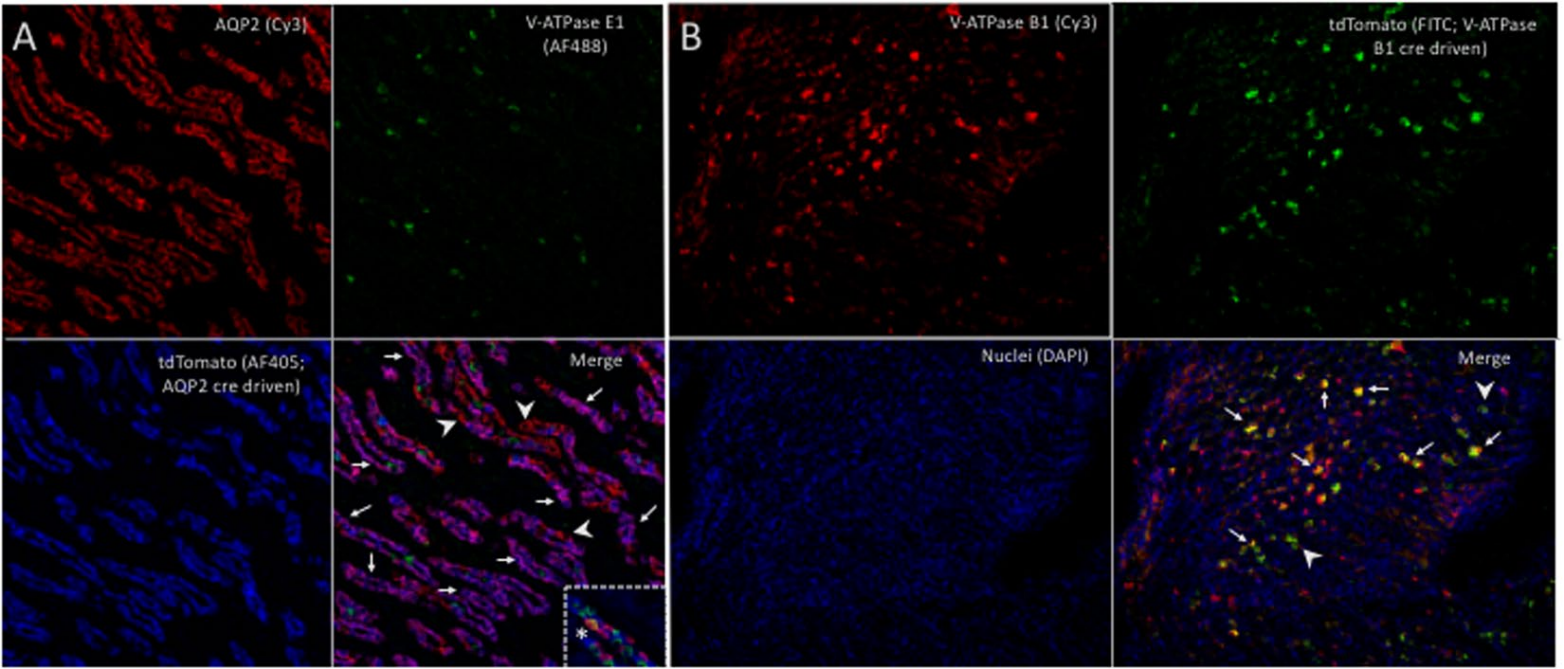
(**A**) Triple immunofluorescence labeling of a kidney section from AQP2-cre^+^tdTomato^+^ (PC reporter) mice showing: AQP2 (red cells, upper left);. V-ATPase E1 (green cells, ICs, upper right); tdTomato (blue cells, lower left) staining in inner medulla. On the merged image (lower right) most AQP2 cells were also positive for tdTomato (purple cells, white arrows) while some were negative for tdTomato (red cells, white arrowheads). The dashed box contains a tubule imaged from another kidney section that contained an isolated cell that was positive for both AQP2 and V-ATPAse E1 (Yellow cell, asterisk). Magnification 40X, confocal microscopy. (**B**) Triple immunofluorescence labeling of a kidney section from V-ATPase B1-cre^+^tdTomato^+^ (IC reporter) mice showing: V-ATPase B1 (red cells, upper left). tdTomato (green cells, upper right) and nuclei (blue, lower left) staining in inner medulla. On the merged image (lower right) most V-ATPase B1 positive cells were also tdTomato positive (yellow cells, white arrows) while a few cells were tdTomato positive but were V-ATPase B1 negative (green cells, white arrowheads). Magnification 20X, immunofluorescent microscopy.

**Figure 5:**
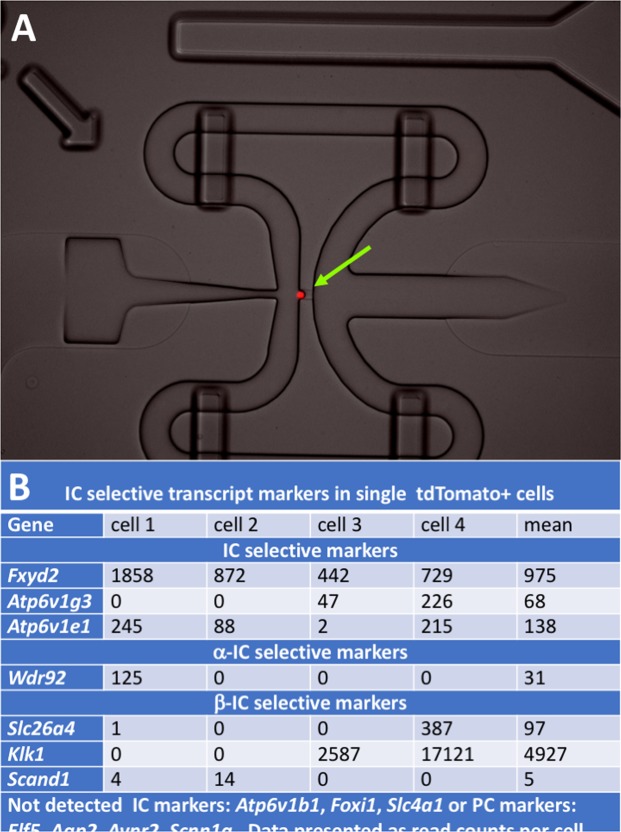
(**A**) Imaging of tdTomato^+^ single intercalated cell (IC) captured on medium size (10–17 µm) IFC plate. Bulk ICs were sorted from IC reporter (V-ATPase-cre x tdTomato) mice with Flow sorting. Cells were pelleted, counted and suspended in Fluidigm cell suspension reagent at 3:2 ratio at 200 cells/µl concentration. Pooled cells were separated to single IC cells on Fluidigm C1 system and imaged under fluorescence microscope at 20X magnification. (**B**) Expression of IC markers. Base on the expression patterns of α-IC and β-IC markers, we estimate that cell 1 was a α-IC while cells 2–3 were β-ICs.

Since *Aqp2* expression (PC cell marker) was present in the enriched IC cells compared to non-IC cells by RNA-Seq (Table 1), we sought to confirm this intriguing finding. When *Aqp2* mRNA expression is overlaid with V-ATPase protein expression, a subset of V-ATPase positive cells (ICs) also appear to express *Aqp2* mRNA (Fig. 3). We counted 155 cells with positive V-ATPase expression from 2 kidneys. 9% (n = 14, 60X magnification) of V-ATPase positive cells also had *Aqp2* mRNA expression. On triple labeling for V-ATPASE E1, AQP2 and tdTomato (expression driven by AQP2 cre promoter), most but not all of the AQP2 positive cells expressed tdTomato (Fig. 4A) indicating that penetrance of cre was good, but not complete in this cre line as well. We also evaluated whether tdTomato (expression driven by *VATPaseB1* promotor) co-labeled with V-ATPase B1 (Fig. 5A–D). Most cells co-labeled with the 2 antibodies but there were some cells that only labelled with tdTomato indicating some “leakiness” of the ATPV1B1-cre (Fig. 4B).

### Single cell IC sequencing pilot study and comparison with recent study by Chen and colleagues

We sequenced 4 intercalated cells on the single cell level and presented the data at https://hpcwebapps.cit.nih.gov/ESBL/Database/IC/index.html/.

The 20 genes with the highest total expression in our single cell IC analysis are presented in Supplemental Data S5, please note this is total expression as opposed to the relative expression presented elsewhere since there were no comparison groups for this analysis. We compared our pooled and single cell data to the 2017 report by Chen and colleagues (Table 5)^22^. For our data, we used the normalized read counts from the IC versus PC comparison for the pooled cell presented in Table 5 because the normalized read counts will change somewhat depending on what other samples are normalized with, and each comparison used datum that was normalized with only the other samples included in that particular comparison.

**Table 5:** Innate immune cell marker comparison.

Gene Symbol	^^^Our pooled IC Mean	^#^Chen *et al*.^22^ pooled IC Mean^^^	^^^Our Single cell IC Mean (n = 4)	^^^Our pooled PC Mean	^#^Chen *et al*.^22^ pooled PC Mean^^^
**Antimicrobial peptide genes**					
*Adm*	161.7	123.6	not detected	40.4	72.6
*Defb1*	8210.5	1973.5	598.2	5797.2	1263.2
*Scg5*	240.6	52.6	26.7	186.0	41.2
**Bacteriostatic nutrient metal scavenger genes**					
*Lcn2*	0.6	14.4	not detected	63.0	120.6
*S100g*	46394.7	4462.4	5267.5	18584.8	1229.6
*S100a11*	557.7	743.2	16	777.8	not listed
**Phagocytosis/endocytosis genes**					
*Cav1*	202.0	104.2	not detected	128.8	497.2
*Cav2*	450.0	388.7	not detected	213.6	759.4
*Rab7*	3710.2	314.7	8.25	2988.7	279.9
**Inflammatory signaling genes**					
*Irf3*	321.7	39.5	98	333.7	47.9
*Il1r1*	113.7	17.6	not detected	83.1	26.9
*Apoe*	1245.8	419.7	422.3	1766.0	2420.0
*Spp1*	18131	3524	1715.7	14659.5	1556.8
*Mmp11*	8	8.6	128.5	not detected	4.7
*Klk1*	124448	6039.2	4927	66958.6	899.8
**Pattern recognition receptor genes**					
*Tlr1*	1.3	6.2	not detected	22.5	13.3
*Tlr2*	nd	8.4	not detected	not detected	26.8
*Tlr3*	130.6	13.4	not detected	138.4	9.5
*Tlr4*	not detected	3.6	not detected	not detected	13.9
*Tril*	4.9	2.3	not detected	25.6	8.88
**Inflammasome genes**					
*Nlrc3*	34.4	0.5	not detected	27.1	0.7
*Naip1*	not detected	0.1	not detected	not detected	not listed

^^^This RNASeq data was obtained using conditional mice expressing tdTomato on V-ATPase-B1 cre (presumed intercalated cells) and AQP2-cre (presumed principal cells). Data presented as mean normalized read count normalized using DEseq2.

^#^This RNASeq data was collected from wild type C57BL/6 mice using DBA lectin (presumed principal cells) and c-kit (presumed intercalated cells) and C1 fluidigm for single cell RNAseq. Data presented as mean TPM (transcripts per kilobase Million) and was acquired from the supplemental data 03 (pooled cells, “All C-Kit(ICs) vs DBA(PCs) from Chen L and *et al*.^22^.

Similar enrichment for PC genes, when pooled PCs were compared to pooled ICs, generally occurred in both datasets^22^. For example, there was 4.1 and 9.7-fold enrichment for *Aqp2* along with 6.6 and 8.3-fold enrichment for *Elf5* in our as well as Chen and colleague’s datasets respectively^22^. IC marker expression was 1.4 to 3.3–fold higher in our pooled ICs vs. pooled PCs analysis but 4.4 to 13.6-fold higher in the Chen and Colleagues analysis^22^. We did identify *Atp6v1e1* subunit expression on the single cell expression level in 4/4 cells and used this gene, along with the IC specific genes, *Fxdy2* and *Atp6v1g3*, to confirm their IC cell status^22,25^. Additionally we did not find PC markers including *Elf5*, *Aqp2*, *Avpr2* or *Scnn1g* in our limited subset of ICs on single cell analysis^22^. An image of the integrated fluidic chip captured a tdTomato expressing single IC (Fig. 5A) and a summary of lineage marker expression is presented in Fig. 5B.

Innate immune gene expression (Table 5) was generally consistent across analysis in pooled ICs and PCs. Both our and Chen and colleague’s analysis revealed that expression for the Antimicrobial peptide (AMP) genes adrenomedullin (*Adm)*, Β-defensin 1 (*Defb1)* and secretogranin 5 (*Scg5)* was present in both pooled ICs and pooled PCs but the mean expression was higher in ICs^22^. Additionally, we were able to identify *Defb1* and *Scg5* in ICs on the single cell level. Lipocalin 2/NGAL (*Lcn2*) had higher expression in PCs than ICs in both pooled cell analysis^22^. The inflammatory signaling genes apolipoprotein E (*ApoE*), secreted phosphoprotein 1/osteopontin (*Spp1*), matrix metallopeptidase (*Mmp11*) and kallikrein-1 (*Klk1*) were identified in ICs at the single cell level. Toll like receptor 3 (*Tlr3*) had the highest expression in the TLR family in pooled ICs in both ours and Chen and colleague’s pooled analysis, but we did not identify this family of genes on the single cell level^22^.

## Discussion

We have completed pooled RNA-Seq of IC and PC along with pilot single cell IC RNA-Seq to lend insight into the biological processes associated with murine renal collecting duct cells. This study complements and expands on our past studies evaluating the transcriptome profile of the collecting duct.

Our RNA-Seq IC component contains overlap compared to the prior proteomic analysis presented by Da Silva and colleagues^26^. Of the 25 genes with the highest differential expression in ICs, several were also reported as enriched in the IC proteomic analysis including Kallikrein 1 (*Klk1)* and Hydroxysteroid 11-beta dehydrogenase 2 (*Hsd11b2)*^26^.

There are also similarities and distinctions when our data is compared to past transcriptome collecting duct expression. Using microdissected rat tubules, Lee and colleagues identified high *Aqp2*, arginine vasopressin receptor 1A (*Avpr1a)*, and *Defb1* expression in the cortical collecting duct and *Aqp2*, *Avpr2*, serum/glucocorticoid regulated kinase 1 (*Sgk1)* and *Defb1* expression in the medullary collecting duct^9^. Similarly, in our analysis *Defb1*, mRNA expression was enriched in both ICs and PCs, akin to our prior targeted RT-PCR analysis of AMP expression in enriched ICs and PCs^14^. The comparison between our data and data generated by Chen and colleagues provides interesting insight into collecting duct function and sequencing capabilities. Both research groups obtained relative enrichment for multiple collecting duct cell type markers. We were able to obtain several IC marker expression in the absence of PC cell marker expression on our single tdTomato + cells (presumed ICs) but not *Atp6v1b1* (Fig. 5). Therefore, our single cell transcriptomics while specific, may not be sensitive. Reasons for this diminished sensitivity may include, low cell number, lower read depth of the sequencing and/or lack of consistent mRNA expression between cells.

Table 1 (Top 25 genes enriched in ICs versus non-ICs) contains some PC associated genes such as *Aqp2* and *Scnn1g*, along with IC genes DMRT like family 2 (*Dmrt2)*, Nuclear protein 1, transcriptional regulator 1 (*Nupr1))* and Arginine vasopressin receptor 1a (Avpr1a*)*; while Table 2 (Top 25 genes enriched in PCs versus non-PCs) highlights historically IC associated genes, cytokine dependent hematopoietic cell linker (*Clnk)*, as well as PC associated gene, mucolipin 3 (*Mcoln3)*^22^. Although enriched (Fig. 1), our pooled collecting duct cell populations may not be absolutely pure and other kidney cell types may contribute to mRNA expression of some genes. This overlap between ICs and PCs might even be more pronounced when these cell types are compared to non-ICs and non-PCs. For example, in the IC versus PC comparison, a limited number of PCs in the ICs sort might result in relatively high PC associated gene expression in enriched ICs compared to the relatively collecting duct poor remainder of the kidney. Therefore, pooled IC and PC cell findings may be generalized to collecting duct cells rather than specific to ICs and PCs. Additionally mRNA expression might not always correlate with protein expression.

Another explanation for overlap between IC and PC genes in our sorted cells is overlap in the transcriptomes of ICs and PCs. For example, the historically PC associated gene *Aqp2* was identified in some ICs using sequencing on the single cell level by Chen and colleagues and by us using *in situ* hybridization (Fig. 3A)^25^. We expectantly had 4.1-fold higher *Aqp2* expression in PCs compared to ICs (adjusted p-value 0.01) but also had this gene come up as enriched in ICs compared to non-ICs (Table 1). Similar results were noted in our targeted RT-PCR collecting duct cell analysis^14^. There is evidence from past studies which suggest that AQP2-expressing cells give rise to ICs^27,28^. We performed *in situ* hybridization for *Aqp2* mRNA expression overlaid with immunostaining for V-ATPase protein expression. Approximately 9% of V-ATPase^+^ cells (presumed ICs) demonstrated *Aqp2* mRNA expression (Fig. 3C). We propose that an intermediary cell type with features of both PCs and ICs is present analogous to the Chen and colleagues which identified *Aqp2* and *Slc4a1* (AE1)/*Slc26a4* (pendrin) dual expressing hybrid cells and Park and colleagues which identified co-expression of AQP2 and V-ATPase B1 at the protein level^22,29^. *Aqp2* expression in our TdTomato expressing cells from the V-ATPase cre-tdTomato mice is higher than would expected from these aforementioned hybrid cells and is likely due to some “leakiness” of the cre (Fig. 4B). Also some *Umod* (a loop of Henle marker) enrichment occurs in the PCs from the tdTomato driven by AQP2 cre, indicating that this cre line also has some overlap with loop cells. Of note Chen *et al*. also reported collecting duct expression of *Umod*, but in ICs rather than PCs, so it is possible that some expression may be intrinsic rather than due to contamination^22^. It is also possible that there may be more overlap between the transcriptomes of ICs and PCs than previously realized and that the functional and structural differences between these cell types are due in part to post translational modifications. We also speculate that the PC enrichment was more efficient than IC enrichment because enrichment of the more prevalent cell type (PCs) conceptually results in less potential for contamination.

In 2011, we proposed that ICs “have a novel role in innate immunity” based on their expression of AMPs^4^. In this study, Ingenuity pathway analysis predicted LPS/IL-1 mediated inhibition of RXR function was the most activated canonical pathway. Decreased retinoid X receptor expression was confirmed to occur 1- hour post experimental murine model of UTI in ICs but not non-ICs. RXR is downregulated indirectly by interferon regulatory factor 3 (*Irf3*) and subsequently suppresses interferon genes^24^. We identified *Irf3* in ICs on the single cell level, and Svanborg and colleagues have shown that *Irf3* SNPs lead to UTI susceptibility^30^. The suppression of *Rxrα* at a 1-hr time point in a non-refluxing C57Bl/6 mouse may indicate an intercalated cell UTI response before a large bacterial burden is able to ascend into the kidneys^31^. Functional consequences of altered RXR expression on the bacterial defense of the kidney and urinary tract will be a key area of future research. Chassin *et al*., reported that renal collecting duct cells bind to uropathogenic E. coli (UPEC) and activate TLR4 dependent and independent pathway^7^. Similarly, we earlier reported that upon UPEC (strain CFT073) challenge *in vitro* and *in vivo*, both ICs and PCs respond quickly by enhancing mRNA expression of anti-microbial peptides^14^. It is of note that in this study, IC associated pathways and LPS interaction are only predicted at the transcriptome level, although we validated the top pathway functionally at mRNA level Nonetheless our analysis will provide a roadmap for future studies that identify the mechanisms by which ICs defend the kidney against bacterial invasion.

We have previously reported that murine and human ICs express innate immune proteins, particularly AMPs in the ribonuclease A and defensin families^6^. This unbiased screen expanded the AMPs known to be expressed by the collecting duct expression. Collecting duct AMPs identified in this study included *Adm Scg5*. *Adm* is a multifunctional protein with direct antimicrobial activity against Staphylococcus and E. coli^32^. *Scg5* is potentially important in the kidney’s anti-bacterial response because secretogranins have been reported to have antimicrobial activity^33^.

The genes with the highest expression in our pilot study of sequenced single ICs (Supplemental Data S5) also lends insight into the innate immune function of these cells. The gene with the highest expression was Metastasis Associated Lung Adenocarcinoma Transcript 1 (*Malat1*). *Malat1* is a conserved long, noncoding RNA that has been demonstrated to be upregulated in LPS activated macrophages and interact with NF-κB subunits to regulate the expression of pro-inflammatory cytokines^34^. Some of the other genes highly expressed in the single ICs also have key innate immune functions. Kallikrein 1(*Klk1*), the 4^th^ most highly expressed gene, is a member of a gene family that has been demonstrated to regulate the antibacterial effects of AMPs by controlling activation of precursor proteins and influencing processing of AMPs to smaller peptides with alternate biological activity^35^. Fms related tyrosine kinase 1 (*Ftl1*), the 6^th^ most highly expressed gene, is also expressed by developing dendritic cells and is involved in Vascular endothelial growth factor mediated dendritic cell inhibition^36,37^. Secreted phosphoprotein 1/osteopontin (*Spp1*), the 8^th^ most highly expressed gene in ICs is also expressed by macrophages, neutrophils and dendritic cells^38^. Prior research indicates that *Spp1* fosters cell adhesion, may facilitate phagocytosis and is involved in migration of neutrophils and dendritic cells^39–41^. High expression of *Malat1*, *Klk1*, *Ftl1* and *Spp1* by ICs indicates that on a transcriptomic level, they contain key components of regulating the antibacterial response.

Our study did have limitations. For example, uromodulin was increased in PCs compared to ICs. This gene should be limited to the Loop of Henle (LOH), therefore it appears that the PC sort, but not IC sort may contain some LOH cells. We speculate that this is from some limitations in our mouse model, specifically some differences between cre induced tdTomato expression and AQP2 expression. Although tdTomato and AQP2 expression co-localized in most cells there were some cells with only AQP2 or tdTomato expression (Fig. 4B). We cannot exclude that cell dissociation has some effect on mRNA expression and/or selects a subset of cells that are able to remain viable through the process. These findings will need to be compared and contrasted to future studies that use other technologies such as laser capture microdissection. Our single cell IC sequencing pilot study evaluated 4 cells, a larger number of cells will be needed to compare expression between subtypes of ICs. Additionally, it is possible that murine and human gene expression may be different. We have seen this with Ribonuclease 7 (RNAse7) which is a potent AMP, is expressed in human ICs but there is no mouse RNAse7 orthologue is known^42^. Lastly, this study was designed to identify genes with differential expression between collecting duct cells and other renal cells. There may be important genes that are highly expressed in the collecting duct that were not highlighted in this study because they are also highly expressed in other nephron segments. Conversely genes with very low expression in one cell population and even lower expression in another cell population in another might be identified as enriched. For example, Hba-2a meets criteria for differentially expressed in ICs versus non ICs (Fig. 1C, left panel) because of expression in some non-ICs and virtually absent expression in ICs.

Our study shows potential limitations of single cell RNA-sequencing as several key cell associated genes were not detected (Fig. 5) this could be due to multiple factors such as sequencing depth, transcript per cell or stability of the single cell transcriptome.

Our study is also different from Chen and colleagues study because our study does not require *in vitro* labeling of cells. Chen and colleagues used a c-kit antibody which may affect the function of c-kit and subsequently cellular proliferation, downstream signal transduction and homologous dimerization^22,43^. Secondly we focused on innate immune signaling where ingenuity analysis predicted LPS/IL-1 mediated inhibition of RXR function among the top canonical pathway of IC function. Lastly we experimentally validated the hybrid phenotype described by Chen *et al*. of V-ATPase^+^Aqp2^+^ cells by combined *in-situ* hybridization and immunofluorescence.

Overall, our study confirms that UPEC leads to decreased *Rxrα* expression in ICs but not ICs consistent with LPS/IL-1 mediated inhibition of RXR function pathway which was the canonical pathway predicted to be most associated with ICs.

## Methods

### Mice

AQP2-cre (B6.Cg-Tg(Aqp2-cre)1Dek/J, (stock no. 006881, Jackson lab, Maine) and VATPaseB1-cre transgenic mice (kindly provided by Dr. Raoul D. Nelson, University of Utah, UT) (in which 7 kb B1 promoter drives the cre expression in ICs) were crossed to tdTomato-l*oxp* homozygous mice (B6.Cg-Gt(ROSA)26Sor^tm9(CAG-tdtomato)Hze/J^ (stock no.007909, Jackson lab, Maine) and genotyped as previously described^14^. All animal research was approved by the Research Institute at Nationwide Children’s Hospital and Indiana University IACUC committees (AR12-00035 and 11333 respectively) and adhered to the “NIH Guide for the Care and Use of Laboratory Animals”. Mice were ~6–10 weeks of age were used. Male mice were used for sequencing experiments and female mice were used for the transurethral infection experiments. Experimental UTIs were induced in female C57Bl/6 mice by transurethral inoculation with 1 × 108 uropathogenic E.coli (UPEC) as previously described. Mice were euthanized and kidney tissue obtained ~1 hour post UTI induction. The UTI induction experiment was completed at Indiana University for 1 mouse, the remainder of murine experiments were completed at Nationwide Children’s Hospital.

### Flowsorting to enrich for renal intercalated and principal cells

Viable renal intercalated cells (IC) and principal cell (PC) were enriched from IC and PC reporter mice as we previously described^14^. This report along with the prior observed function of collecting duct as long as 8 hour following euthanasia of the animal supports the stable properties of collecting duct cells^14,22,44^. Briefly, kidneys were harvested in cold PBS and immediately transferred to Accumax solution (cat.no. AM-105, Innovative Cell Technologies, CA) placed in C tubes (Miltenyi Biotec, CA). Cells were then rapidly disrupted to single cell suspension using gentleMACS dissociator (Miltenyi Biotec, CA) within 2 minutes. Renal resident immune cells separated by incubation with anti-mouse CD45-APC (Cat.no.17-0451, clone 30-F-11, eBiosciences, CA) for 30 min at 4 °C. Doublets, clumps and dead cells were removed from the cell suspension and viable cells were obtained as previously described^14^. Enriched ICs and PCs came from three biological replicates/sorts, each from a distinct mouse

### SMARTer stranded total RNA-sequencing

Following DNase treatment and cleanup of RNA from isolated cells using Zymo RNA cleanup and concentrator kit (Zymo research, CA) quality assessment was performed on Agilent 2100 bioanalyzer with RNA Pico chip kit (Agilent Technologies, CA). RNA integrity score ≥6 was used for cDNA library preparation from each sample. 10 ng of total RNA was used to generate RNA-Seq libraries using SMARTer stranded total RNA-Seq kit-Pico input mammalian kit (Clontech Laboratories Inc., CA). Briefly, total RNA was converted to cDNA using SMART^®^ (Switching Mechanism at 5′ end of RNA template) and locked nucleic acid (LNA) technology included as part of the template-switching oligo (TSO). Ribosomal cDNA (originating from rRNA) was then cleaved by ZapR in the presence of mammalian-specific R-probes. The library fragments originating from non-rRNA molecules were enriched via PCR amplification. Quality of the libraries were assessed via Agilent 2100 Bioanalyzer using DNA High Sensitivity Chip kit, and quantified using Kappa SYBR^®^Fast qPCR kit (KAPA Biosystems, MA). 151 bp sequence paired end reads were generated using the Illumina HiSeq 4000 platform (Illumina, CA).

### RNA-Seq data analysis

On average, 50 million paired-end 151 bp RNA-Seq reads were generated for each sample (the range was 34 to 84 million). Each sample was aligned to the GRCm38.p4 assembly of the mouse reference from NCBI using version 2.5.0c of the RNA-Seq aligner STAR^45^. Transcript features were identified from the GFF file provided with the GRCm38.p4 assembly from NCBI. Feature coverage counts were calculated using HTSeq (http://www-huber.embl.de/users/anders/HTSeq/doc/count.html). The raw RNA-Seq gene expression data was normalized and post-alignment statistical analyses performed using DESeq2 and custom analysis scripts written in R. Differentially expressed features were calculated using DESeq2^46^. Maximum-likelihood estimates (MLE) were used to determine log2 fold change which was converted to fold change^46^. The differential expression results (including the p-values) we report for all comparisons were calculated by DESeq2, which uses “the procedure of Benjamini and Hochberg” for pooled cell analysis and for multiple testing adjustment, as specified in the DESeq2^46^.

Comparisons of gene expression and associated statistical analysis were made between different conditions of interest using the normalized read counts. All fold change values are expressed as test condition/control condition, where values less than one are denoted as the negative of its inverse (note that there will be no fold change values between −1 and 1, and that the fold changes of “1” and “−1” represent the same value).

Comparison groups consisted of ICs versus non-ICs, PCs versus non-PCs and ICs versus PCs. The “IC vs. NIC” or “PC vs. NPC” worksheet shows significant differentially expressed features for the comparison of the IC group versus the NIC group. Innate immune genes were identified by keyword search (Supplemental Data S3) of the RNA-Seq results. Biological relevance of identified transcripts was evaluated using Ingenuity Pathway Analysis (IPA; Ingenuity Systems, Redwood City, CA. http://www.ingenuitypathway.org; version 14197757). Figures of canonical pathways and upstream regulators were made with the Ingenuity Pathway Analysis Software. Only transcripts whose absolute value of fold change is ≥2 and have an adjusted p value ≤ 0.0.05 were included in the Ingenuity pathway analysis and development of Tables 1–3.

### *Aqp2* mRNA *in-situ* hybridization

*Aqp2* mRNA was visualized in 4μm paraffin embedded mouse kidney sections using the QuantiGene ViewRNA ISH Tissue Assay according to the manufacturer’s protocol (Affymetrix, Santa Clara, CA). Prehybridization conditions were optimized with 5 minutes of incubation in pretreatment solution (90 °C) and 5 minutes of treatment with protease QF (40 °C). Sections were hybridized for 3 hours (40 °C) with a viewRNA Probe Set designed against *Aqp2* mRNA. The probes were then amplified (Pre Amplifier and Amplifier molecules), conjugated to alkaline phosphatase (Label Probe 1-AP), and bound with Fast Red Substrate. Finally, sections were counterstained with hematoxylin and imaged with the EVOS FL Auto Cell Imaging System (Life Technologies, Grand Island, NY). *In-situ* hybridized slides were incubated with anti-mouse V-ATPase E1 antibody (Sigma, St. Louis, MO) for 90 min at room temperature and then incubated with anti-chicken AF488 conjugated IgG secondary antibody (Jackson Immunoresearch, PA) and mounted with DAPI nuclear stain (Vector Lab, CA). The V-ATPase E1 subunit, like the B1 subunit is specific for ICs in murine kidneys^16^. Slides were visualized under Keyence BZ9000 microscope (Keyence, Japan).

### Targeted RT-PCR

Selected genes found to have higher relative expression in IC vs NIC or PC vs NPC RNA seq results were validated using real-time PCR. To do this IC and PC cells were sorted from respective reporter mice as previously described. RNA was prepared, with RNeasy plus micro kit (Qiagen, CA), quality was validated with Agilent Bioanalyzer and reverse transcribed to cDNA with high capacity reverse transcription kit (Applied Biosystems, CA) and amplified with specific primer sets (Sigma, MO). ΔΔCT method was used to calculate fold change in gene expression as previously described^1^.

### Functional validation of LPS-IL-1 mediated inhibition of RXRA function in Intercalated vs non-intercalated cells of UPEC infected mice

To functionally validate the top IC pathway (LPS/IL-1 mediated inhibition of RXR function) identified by ingenuity analysis, V-ATPase-cre^+^ Tdt^+^ (IC reporter) mice were transurethrally infected with 1 × 10^8^ CFU UPEC or saline. After 1 hr, mice were sacrificed and kidney cell suspension was prepared. Tdt^+^ IC and Tdt^−^ non-IC cells were flowsorted as previously shown and *Rxra* mRNA expression was measured in sorted cells. Primer sequence of *Rxra* were as follows; *Rxra* Forward: TAACAGAGCTGGTGTCTAAG, Reverse *Rxra*: TTAGAGTCAGGGTTGAACAG.

### Immunofluorescence

4 µm paraffin embedded kidney sections from AQP2-cre^+^/tdTomato^*flox*^ and V-ATPase B1-cre^+^/tdTomato^*flox*^ mice were stained with AQP2, V-ATPase B1, V-ATPase E1, and/or tdTomato antibodies to visualize the specificity of cre recombination. *AQP2-cre mice*: In this mice, to visualize AQP2, anti-mouse AQP2 antibody (Santa Cruz, CA), 1:200 dilution, 1 hr at 4 °C), to visualize V-ATPase, anti-mouse V-ATPase E1 antibody (1:500 dilution, Sigma, MO, overnight at 4 °C) were used, to visualize tdTomato, anti-RFP antibody (Rockland antibodies, PA) at 1:200 dilution was incubated overnight at 4 °C. Secondary antibody for AQP2 was Cy3 conjugated anti-goat IgG (1:300 dilution), for V-ATPase, AF488 conjugated anti-chicken IgG (1:300 dilution), for tdTomato, AF405 conjugated anti-rabbit IgG (1:300 dilution). Sections were imaged with Zeiss LSM 510 confocal microscope (Oberkochen, Germany). *V-ATPase-cre mice*: In this mice, to visualize, V-ATPase, goat anti-mouse V-ATPase B1/B2 antibody (E20) (sc21209, 1:50 dilution, Santa Cruz, CA), to visualize tdTomato, anti-RFP-FITC conjugated antibody (ab34764, 1:200 dilution, abcam, MA) were incubated overnight at 4 °C. Secondary antibody for V-ATPase was Cy3 conjugated anti-goat IgG (1:300 dilution) was used for 1 hr at room temperature. All secondary antibodies were purchased from Jackson Immunoresearch, PA. Sections were visualized under Keyence BZ-9000 microscope (Keyence, Japan).

### Single cell capture, cDNA generation and sequencing

TdTomato^+^ cells flowsorted from IC reporter mice as described previously. Briefly kidneys were harvested and immediately transferred to Accumax solution (Innovative Cell technology, CA) and single cell suspension was rapidly prepared with GentleMACS dissociator (Miltenyi Biotec, CA) with Lung program 40 second and spleen program 60 seconds. TdTomato^+^ cells were flowsorted. Sorted cells were pelleted and suspended to a final concentration of 200 cells/µl in sterile PBS. Cells were then combined with C1 Fluidigm suspension reagent at 3:2 ratio and loaded on a medium sized (for 10–17 µm diameter cells) Fluidigm IFC for single cell capture on the C1 system. Each IFC chamber was visually inspected under microscope to find wells with individual single cells and to avoid doublets or damaged cells (Fig. 5). Each cell was then washed, lysed and reverse transcribed using oligo dT primer and amplified by PCR automatically on C1 with SMART-seq v4 ultra low input RNA kit (Clontech, CA). cDNAs were harvested from the outlets of IFC and quantified by Qubit 2.0 fluorometer and DNA size was determined by Agilent 2100 Bioanalyzer using High-sensitivity DNA kit (Agilent, CA). RNA-Seq library was prepared with an input amount of 1 ng of cDNA from the Fluidigm Clontech step using the Nextera XT DNA library preparation kit (Illumina, CA) and sequenced on the Illumina Hiseq. 4000 platform (Illumina, CA) to a depth of 10 million paired reads per cell.

## Supplementary information

Supplemental Data 

## References

[1] Hasler U (2002). Long term regulation of aquaporin-2 expression in vasopressin-responsive renal collecting duct principal cells. The Journal of biological chemistry.

[2] Brown D, Hirsch S, Gluck S (1988). An H+-ATPase in opposite plasma membrane domains in kidney epithelial cell subpopulations. Nature.

[3] Hains, D. S. *et al*. Carbonic anhydrase 2 deficiency leads to increased pyelonephritis susceptibility. *American journal of physiology. Renal physiology*, 10.1152/ajprenal.00344.2014 (2014). 10.1152/ajprenal.00344.2014 PMC4187042 25143453

[4] Spencer JD (2011). Ribonuclease 7 is a potent antimicrobial peptide within the human urinary tract. Kidney Int.

[5] Spencer JD (2012). Human alpha defensin 5 expression in the human kidney and urinary tract. PloS one.

[6] Paragas N (2014). alpha-Intercalated cells defend the urinary system from bacterial infection. J Clin Invest.

[7] Chassin C (2006). Renal collecting duct epithelial cells react to pyelonephritis-associated Escherichia coli by activating distinct TLR4-dependent and -independent inflammatory pathways. J Immunol.

[8] St-Pierre C (2013). Transcriptome sequencing of neonatal thymic epithelial cells. Scientific reports.

[9] Lee JW, Chou CL, Knepper MA (2015). Deep Sequencing in Microdissected Renal Tubules Identifies Nephron Segment-Specific Transcriptomes. J Am Soc Nephrol.

[10] Robert-Nicoud M (2001). Transcriptome of a mouse kidney cortical collecting duct cell line: effects of aldosterone and vasopressin. Proceedings of the National Academy of Sciences of the United States of America.

[11] Ryazanova MA (2016). The gene-expression profile of renal medulla in ISIAH rats with inherited stress-induced arterial hypertension. BMC genetics.

[12] Miller RL (2005). V-ATPase B1-subunit promoter drives expression of EGFP in intercalated cells of kidney, clear cells of epididymis and airway cells of lung in transgenic mice. American journal of physiology. Cell physiology.

[13] Nelson RD (1998). Expression of an AQP2 Cre recombinase transgene in kidney and male reproductive system of transgenic mice. The American journal of physiology.

[14] Saxena, V. *et al*. Cell specific qRT-PCR of renal epithelial cells reveals a novel innate immune signature in murine collecting duct. *American journal of physiology. Renal physiology*, ajprenal00512 02016, 10.1152/ajprenal.00512.2016 (2017). 10.1152/ajprenal.00512.2016 PMC6230735 28468965

[15] Azroyan A (2015). Renal intercalated cells sense and mediate inflammation via the P2Y14 receptor. PLoS One.

[16] Vedovelli L (2013). Altered V-ATPase expression in renal intercalated cells isolated from B1 subunit-deficient mice by fluorescence-activated cell sorting. American journal of physiology. Renal physiology.

[17] Kurth I (2006). The forkhead transcription factor Foxi1 directly activates the AE4 promoter. Biochem J.

[18] Yu MJ (2009). Systems-level analysis of cell-specific AQP2 gene expression in renal collecting duct. Proc Natl Acad Sci USA.

[19] Rubera I (2003). Collecting duct-specific gene inactivation of alphaENaC in the mouse kidney does not impair sodium and potassium balance. J Clin Invest.

[20] Legouis D (2015). *Ex vivo* analysis of renal proximal tubular cells. BMC Cell Biol.

[21] Hoyer JR, Sisson SP, Vernier RL (1979). Tamm-Horsfall glycoprotein: ultrastructural immunoperoxidase localization in rat kidney. Lab Invest.

[22] Chen L (2017). Transcriptomes of major renal collecting duct cell types in mouse identified by single-cell RNA-seq. Proc Natl Acad Sci USA.

[23] Roszer T, Menendez-Gutierrez MP, Cedenilla M, Ricote M (2013). Retinoid X receptors in macrophage biology. Trends Endocrinol Metab.

[24] Ma F (2014). Retinoid X receptor alpha attenuates host antiviral response by suppressing type I interferon. Nat Commun.

[25] Paunescu TG (2013). Vasopressin induces apical expression of caveolin in rat kidney collecting duct principal cells. Am J Physiol Renal Physiol.

[26] Da Silva N (2010). Proteomic analysis of V-ATPase-rich cells harvested from the kidney and epididymis by fluorescence-activated cell sorting. Am J Physiol Cell Physiol.

[27] Wu H (2013). Aqp2-expressing cells give rise to renal intercalated cells. J Am Soc Nephrol.

[28] Trepiccione F (2016). A fate-mapping approach reveals the composite origin of the connecting tubule and alerts on “single-cell”-specific KO model of the distal nephron. Am J Physiol Renal Physiol.

[29] Park J (2018). Single-cell transcriptomics of the mouse kidney reveals potential cellular targets of kidney disease. Science.

[30] Fischer H (2010). Pathogen specific, IRF3-dependent signaling and innate resistance to human kidney infection. PLoS Pathog.

[31] Murawski IJ (2010). The C3H/HeJ inbred mouse is a model of vesico-ureteric reflux with a susceptibility locus on chromosome 12. Kidney Int.

[32] Allaker RP (2006). Mechanisms of adrenomedullin antimicrobial action. Peptides.

[33] Shooshtarizadeh P (2010). The antimicrobial peptides derived from chromogranin/secretogranin family, new actors of innate immunity. Regul Pept.

[34] Zhao G, Su Z, Song D, Mao Y, Mao X (2016). The long noncoding RNA MALAT1 regulates the lipopolysaccharide-induced inflammatory response through its interaction with NF-kappaB. FEBS Lett.

[35] Yamasaki K (2006). Kallikrein-mediated proteolysis regulates the antimicrobial effects of cathelicidins in skin. FASEB J.

[36] Dikov MM (2005). Differential roles of vascular endothelial growth factor receptors 1 and 2 in dendritic cell differentiation. J Immunol.

[37] Laxmanan S (2005). Vascular endothelial growth factor impairs the functional ability of dendritic cells through Id pathways. Biochem Biophys Res Commun.

[38] Wang KX, Denhardt DT (2008). Osteopontin: role in immune regulation and stress responses. Cytokine Growth Factor Rev.

[39] McKee MD, Nanci A (1996). Secretion of Osteopontin by macrophages and its accumulation at tissue surfaces during wound healing in mineralized tissues: a potential requirement for macrophage adhesion and phagocytosis. Anat Rec.

[40] Koh A (2007). Role of osteopontin in neutrophil function. Immunology.

[41] Weiss JM (2001). Osteopontin is involved in the initiation of cutaneous contact hypersensitivity by inducing Langerhans and dendritic cell migration to lymph nodes. J Exp Med.

[42] Cho S, Beintema JJ, Zhang J (2005). The ribonuclease A superfamily of mammals and birds: identifying new members and tracing evolutionary histories. Genomics.

[43] Liang J (2013). The C-kit receptor-mediated signal transduction and tumor-related diseases. Int J Biol Sci.

[44] Grantham JJ, Burg MB (1966). Effect of vasopressin and cyclic AMP on permeability of isolated collecting tubules. Am J Physiol.

[45] Dobin A (2013). STAR: ultrafast universal RNA-seq aligner. Bioinformatics.

[46] Love MI, Huber W, Anders S (2014). Moderated estimation of fold change and dispersion for RNA-seq data with DESeq2. Genome Biol.

