# Publisher Correction: Whole Transcriptome Analysis of Renal Intercalated Cells Predicts Lipopolysaccharide Mediated Inhibition of Retinoid X Receptor alpha Function

**DOI:** 10.1038/s41598-020-60599-x

**Published:** 2020-03-17

**Authors:** Vijay Saxena, James Fitch, John Ketz, Peter White, Amy Wetzel, Melinda A. Chanley, John D. Spencer, Brian Becknell, Keith R. Pierce, Sam W. Arregui, Raoul D. Nelson, George J. Schwartz, Victoria Velazquez, Logan A. Walker, Xi Chen, Pearlly Yan, David S. Hains, Andrew L. Schwaderer

**Affiliations:** 1 Indiana University School of Medicine, Riley Children’s Hospital, Indianapolis, Indiana United States; 2 The Institute for Genomic Medicine, Nationwide Children’s Hospital, Columbus, Ohio United States; 3 The Research Institute at Nationwide Children’s, Center for Clinical and Translational Research, Columbus, Ohio, and College of Medicine, Ohio State University, Columbus, Ohio United States; 4 Department of Pediatrics, College of Medicine, The Ohio State University, Columbus, Ohio United States; 5 Innate Immunity Translational Research Center, Children’s Foundation Research Institute at Le Bonheur Children’s Hospital, Memphis, Tennessee United States; 6 Division of Nephrology, Department of Pediatrics, University of Utah, Salt Lake City, Utah United States; 7 University of Rochester Medical Center, School of Medicine and Dentistry, Rochester, New York United States; 8 Research Institute at Nationwide Children’s Hospital Flow Cytometry Core Laboratory, Columbus, Ohio United States; 9 Department of Physics, College of Arts and Sciences, The Ohio State University, Columbus, Ohio United States; 10 Genomics Shared Resource, The Ohio State University Comprehensive Cancer Center, Columbus, Ohio United States; 11 Division of Hematology, Department of Internal Medicine, The Ohio State University Wexner Medical Center, Columbus, Ohio United States

Correction to: *Scientific Reports* 10.1038/s41598-018-36921-z, published online 24 January 2019

The original version of this Article contained multiple errors.

In the Abstract,

“IC associated genes included the innate interleukin 1 receptor, type 1 and the antimicrobial peptide(AMP) adrenomedullin.”

now reads:

“IC associated genes included the innate interleukin 1 receptor, type 1 and the antimicrobial peptide (AMP) adrenomedullin.”

In the legend of Figure 2,

“**(B)** IC specific inhibition of R*xrα* mTNA expression.”

now reads:

“**(B)** IC specific inhibition of R*xrα* mRNA expression.”

In addition, there was a typographical error in the ‘Results’ section under the subsection, ‘Functional evaluation of LPS/IL-1 mediated inhibition of RXR function in IC versus non-ICs’, where:

“One of its member, RXR which is a key nuclear receptor in metabolic processes has been shown to be down regulated during viral infections and protect host^24^.”

now reads:

“One of its member, RXR *α* which is a key nuclear receptor in metabolic processes has been shown to be down regulated during viral infections and protect host^24^.”

Furthermore, the p-Adjusted value for *Prkar1b* was missing from Table 2.

The original Article also contained a truncated Figure 5.

There was an additional typographical error in the link in the ‘Results’ section where:

“https://hpcwebapps.cit.nih.gov/ESBL/Database/IC/index.html/”

now reads:

“https://hpcwebapps.cit.nih.gov/ESBL/Database/IC/index.html”

Finally, there was also a typographical error in the ‘Discussion’ section, where:

“Overall, our study confirms that UPEC leads to decreased *Rxrα* expression in ICs but not ICs consistent with LPS/IL-1 mediated inhibition of RXR function pathway which was the canonical pathway predicted to be most associated with ICs.”

now reads:

“Overall, our study confirms that UPEC leads to decreased *Rxrα* expression in ICs but not non-ICs consistent with LPS/IL-1 mediated inhibition of RXR function pathway which was the canonical pathway predicted to be most associated with ICs.”

These errors have now been corrected in the PDF and HTML versions of the Article.

