# Supplementary material for: Whole Transcriptome Analysis of Renal Intercalated Cells Predicts Lipopolysaccharide Mediated Inhibition of Retinoid X Receptor alpha Function
Source: Sci Rep. 2019 Jan 24;9:545. doi: 10.1038/s41598-018-36921-z (PMC6345901; doi:10.1038/s41598-018-36921-z)
Supplement: Supplementary file 1 — Supplemental Data [file 41598_2018_36921_MOESM1_ESM.pdf]

Supplemental information

Whole Transcriptome Analysis of Renal Intercalated Cells Predicts Lipopolysaccharide Mediated Inhibition of Retinoid X Receptor alpha Function

Vijay Saxena, James Fitch, John Ketz, Peter White, Amy Wetzel, Melinda A Chanley, John D Spencer, Brian Becknell, Keith R Pierce, Sam W Arregui, Raoul D Nelson, George J Schwartz, Victoria Velazquez, Logan A. Walker, Xi Chen, Pearlly Yan, David S Hains, Andrew L Schwaderer

S1

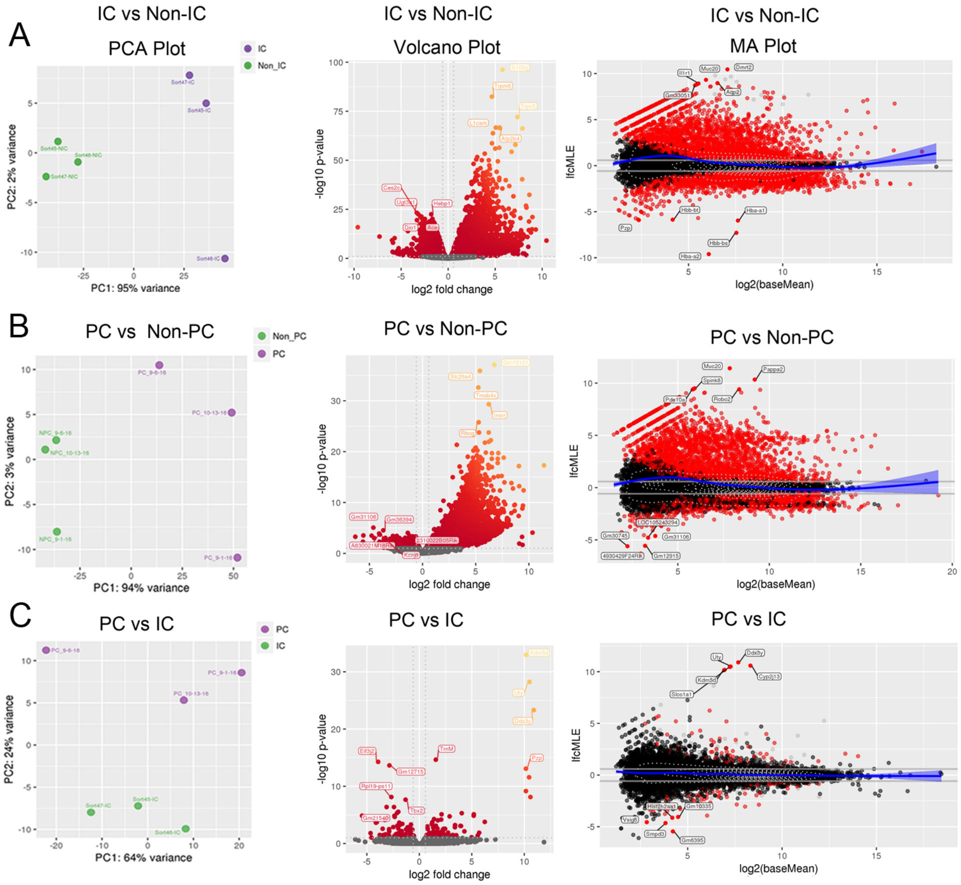
A
IC vs Non-IC
PCA Plot
IC
Non_IC
PC2: 2% variance
PC1: 95% variance
IC vs Non-IC
Volcano Plot
-log10 p-value
log2 fold change
IC vs Non-IC
MA Plot
lfcMLE
log2(baseMean)
B
PC vs Non-PC
Non_PC
PC
PC2: 3% variance
PC1: 94% variance
PC vs Non-PC
-log10 p-value
log2 fold change
PC vs Non-PC
lfcMLE
log2(baseMean)
C
PC vs IC
PC
IC
PC2: 24% variance
PC1: 64% variance
PC vs IC
-log10 p-value
log2 fold change
PC vs IC
lfcMLE
log2(baseMean)

S2

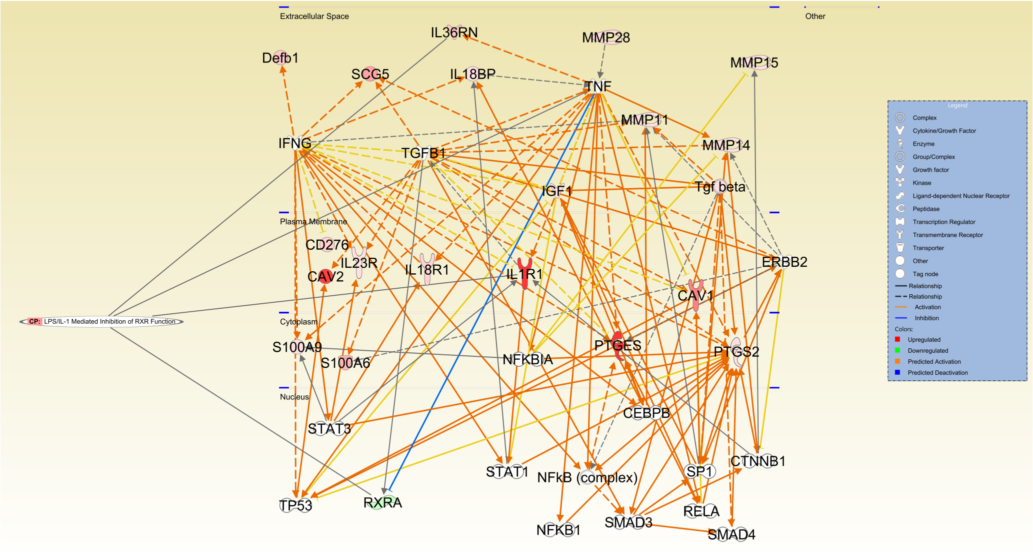

Extracellular Space
Other
Plasma Membrane
Cytoplasm
Nucleus
IL36RN
MMP28
Defb1
SCG5
IL18BP
TNF
MMP15
MMP11
IFNG
TGFB1
MMP14
IGF1
Tgf beta
CD276
IL23R
CAV2
IL18R1
IL1R1
ERBB2
CAV1
CP: LPS/IL-1 Mediated Inhibition of RXR Function
S100A9
S100A6
NFKBIA
PTGES
PTGS2
CEBPB
STAT3
STAT1
NFkB (complex)
SP1
CTNNB1
TP53
RXRA
RELA
NFKB1
SMAD3
SMAD4
Legend
Complex
Cytokine/Growth Factor
Enzyme
Group/Complex
Growth factor
Kinase
Ligand-dependent Nuclear Receptor
Peptidase
Transcription Regulator
Transmembrane Receptor
Transporter
Other
Tag node
Relationship
Relationship
Activation
Inhibition
Colors:
Upregulated
Downregulated
Predicted Activation
Predicted Deactivation

S3

ID	Description	EntrezID	Class	PC.vs.IC_ShrunkenLog2FC	PC.vs.IC_MLELog2FC	PC.vs.IC_pVal	PC.vs.IC_pAdj	PC_Mean	IC_Mean
Nlrc3	NLR family, CARD domain containing 3	268857	protein_coding	-0.238042585	-0.32837145	0.440208098	0.999225654	27.08	34.04
Nlrc5	NLR family, CARD domain containing 5	434341	protein_coding	0.380530721	0.54916209	0.232247580	0.948887454	40.88	27.59
Nlrp5-ps	NLR family, pyrin domain containing 5, pseudogene	100417675	misc_RNA	0.150533751	0.676249917	0.603436181	0.999225654	9.98	6.43
Nlrp6	NLR family, pyrin domain containing 6	101613	protein_coding	0.034996815	0.091423471	0.916949431	0.999225654	545.53	511.93
Nlrx1	NLR family member X1	270151	protein_coding	0.484836628	0.606876565	0.079805938	0.758494081	129.57	84.32
Nod1	nucleotide-binding oligomerization domain containing 1	107607	protein_coding	0.197646388	0.251107682	0.484180744	0.999225654	132.34	111.36
Nod2	nucleotide-binding oligomerization domain containing 2	257632	protein_coding	0.073124246	0.527939471	0.759161540		4.8	3.22
Aim1	absent in melanoma 1	11630	protein_coding	0.191403819	0.227121655	0.446357358	0.999225654	401.91	342.96
Tlr1	toll-like receptor 1	21897	protein_coding	0.939578647	3.986969602	0.003026807	0.169723242	22.5	1.3
Tlr12	toll-like receptor 12	384059	protein_coding	-0.031830975	-0.18766233	0.902324403	0.999225654	8.14	9.2
Tlr3	toll-like receptor 3	142980	protein_coding	0.05679925	0.083851558	0.860359366	0.999225654	138.43	130.57
Tril	TLR4 interactor with leucine-rich repeats	66873	protein_coding	0.553914099	2.341891237	0.067082855	0.72242614	25.59	4.87
Pin1	protein (peptidyl-prolyl cis/trans isomerase) NIMA-interacting 1	23988	protein_coding	-0.808255551	-0.886257271	3.60898E-05	0.007487602	183.27	338.9
Il10rb	interleukin 10 receptor, beta	16155	protein_coding	0.088300753	0.158924135	0.796893818	0.999225654	149.46	133.34
Il11ra1	interleukin 11 receptor, alpha chain 1	16157	protein_coding	-0.131571885	-0.159763967	0.617105748	0.999225654	536.4	600.3
Il12rb2	interleukin 12 receptor, beta 2	16162	protein_coding	0.39612446	1.210544904	0.223526958	0.946437698	8.8	3.54
Il13ra1	interleukin 13 receptor, alpha 1	16164	protein_coding	0.606606295	0.683783683	0.005475612	0.235236099	303.52	188.49
Il15	interleukin 15	16168	protein_coding	0.168635267	0.223517926	0.570425427	0.999225654	90.24	77.18
Il15ra	interleukin 15 receptor, alpha chain	16169	protein_coding	0.038157653	0.047881499	0.890783553	0.999225654	198.54	191.72
Il17ra	interleukin 17 receptor A	16172	protein_coding	-0.11567222	-0.619668446	0.667517500	0.999225654	5.95	8.94
Il17rb	interleukin 17 receptor B	50905	protein_coding	0.891268907	1.153031073	0.001967254	0.148804344	63.53	28.97
Il17rc	interleukin 17 receptor C	171095	protein_coding	0.220812659	0.281985569	0.437647627	0.999225654	84.98	70.15
Il17rd	interleukin 17 receptor D	171463	protein_coding	0.581219312	0.795342889	0.057551797	0.690193841	340.77	196.76
Il17re	interleukin 17 receptor E	57890	protein_coding	0.02300714	0.034419396	0.943597874	0.999225654	92.03	89.69
Il18	interleukin 18	16173	protein_coding	0.237534362	1.286316822	0.381909700	0.999225654	8.57	3.45
Il18bp	interleukin 18 binding protein	16068	protein_coding	0.100054834	0.171603489	0.768797573	0.999225654	65.01	58.19
Il18r1	interleukin 18 receptor 1	16182	protein_coding	-0.358872785	-0.951698833	0.284224051	0.977155712	9.95	18.94
Il1f5	interleukin 1 family, member 5 (delta)	54450	protein_coding	-0.448268862	-1.879795247	0.135020810	0.869945825	8.39	30.87
Il1r1	interleukin 1 receptor, type I	16177	protein_coding	-0.310087213	-0.452461808	0.333538495	0.997759559	83.09	113.68
Il1rap	interleukin 1 receptor accessory protein	16180	protein_coding	0.350363986	0.532602108	0.284761817	0.977155712	67.35	46.3
Il1rl2	interleukin 1 receptor-like 2	107527	protein_coding	-0.317065388	-0.609221267	0.358011491	0.999225654	10.34	15.76
Il20rb	interleukin 20 receptor beta	213208	protein_coding	0.076548133	0.577294326	0.745323245	0.999225654	6.6	4.48
Il22ra1	interleukin 22 receptor, alpha 1	230828	protein_coding	0.034771374	0.090246207	0.917567547	0.999225654	42.75	39.83
Il33	interleukin 33	77125	protein_coding	-0.134460424	-0.815492585	0.602612778		3.14	5.45
Il34	interleukin 34	76527	protein_coding	0.23072887	0.969196249	0.435907490	0.999225654	20.07	10.35
Il4ra	interleukin 4 receptor, alpha	16190	protein_coding	0.064435574	0.074404553	0.784201292	0.999225654	241.34	229.13
Il5ra	interleukin 5 receptor, alpha	16192	protein_coding	0.057662656	0.161425583	0.861668727	0.999225654	56.11	50.07
Il6ra	interleukin 6 receptor, alpha	16194	protein_coding	0.197925803	1.212632289	0.443761420	0.999225654	13.06	5.48
Il6st	interleukin 6 signal transducer	16195	protein_coding	0.190727395	0.207210519	0.307311574	0.988427002	1419.21	1229.45
Il7	interleukin 7	16196	protein_coding	-0.018724688	-0.083240167	0.948066792	0.999225654	6.98	7.26
Tgfbr1	transforming growth factor, beta receptor I	21812	protein_coding	0.006047428	0.00740435	0.981939213	0.999444767	689.07	686.22
Tgfbr2	transforming growth factor, beta receptor II	21813	protein_coding	0.170819371	0.192283048	0.432073717	0.999225654	427.84	373.88
Tgfbr3	transforming growth factor, beta receptor III	21814	protein_coding	-0.008294049	-0.013222608	0.980181310	0.999444767	264.3	266.47
Tgfbr3l	transforming growth factor, beta receptor III-like	100044509	protein_coding	0.077141437	0.134028199	0.821123480	0.999225654	44.49	40.95
Ifnar1	interferon (alpha and beta) receptor 1	15975	protein_coding	0.372609874	0.399772673	0.031969347	0.539616992	512.21	388.88
Ifnar2	interferon (alpha and beta) receptor 2	15976	protein_coding	0.152436524	0.17584333	0.515900406	0.999225654	145.25	127.73
Ifngr1	interferon gamma receptor 1	15979	protein_coding	0.030666744	0.033212235	0.867393398	0.999225654	237.66	232.35
Ifngr2	interferon gamma receptor 2	15980	protein_coding	0.226368556	0.265500018	0.355056889	0.999225654	149.27	123.86
Ifnlr1	interferon lambda receptor 1	242700	protein_coding	0.129401522	0.221927742	0.704013278	0.999225654	42.73	36.06
P2rx3	purinergic receptor P2X, ligand-gated ion channel, 3	228139	protein_coding	-0.406605387	-0.939019382	0.235042266	0.95031148	9.5	17.92
P2rx4	purinergic receptor P2X, ligand-gated ion channel 4	18438	protein_coding	0.354538776	0.396787146	0.095855199	0.800135973	303.98	231.13
P2rx7	purinergic receptor P2X, ligand-gated ion channel, 7	18439	protein_coding	0.062319522	0.462620451	0.791653267	0.999225654	6.83	4.83
P2ry1	purinergic receptor P2Y, G-protein coupled 1	18441	protein_coding	-0.471277412	-0.626539862	0.114018275	0.835884608	35.42	54.28
P2ry14	purinergic receptor P2Y, G-protein coupled, 14	140795	protein_coding	-0.615847097	-1.733225706	0.064112269	0.708607692	8.32	27.19
P2ry2	purinergic receptor P2Y, G-protein coupled 2	18442	protein_coding	0.575163216	0.982607518	0.090643893	0.787804462	45.13	22.72
Cd14	CD14 antigen	12475	protein_coding	0.190358595	0.774688815	0.524268407	0.999225654	10.42	6.08
Cd151	CD151 antigen	12476	protein_coding	0.064444842	0.079449269	0.811489043	0.999225654	419.74	397.94
Cd164	CD164 antigen	53599	protein_coding	0.099380051	0.11181767	0.647056563	0.999225654	1150.49	1064.75
Cd164l2	CD164 sialomucin-like 2	69655	protein_coding	-0.464771291	-0.850351775	0.176219949	0.915910448	79.57	143.65
Cd200	CD200 antigen	17470	protein_coding	-0.169431704	-0.281256408	0.616081687	0.999225654	130.54	158.77
Cd247	CD247 antigen	12503	protein_coding	-0.025123806	-0.243783048	0.905060626		3.07	3.64
Cd248	CD248 antigen, endosialin	70445	protein_coding	-0.633953281	-2.585366695	0.038834174	0.591803469	2.37	14.29
Cd24a	CD24a antigen	12484	protein_coding	0.364589812	0.517468907	0.247001706	0.958974344	1612.74	1127.23
Cd274	CD274 antigen	60533	protein_coding	0.184920649	0.243837425	0.530910514	0.999225654	43.93	37.66
Cd276	CD276 antigen	102657	protein_coding	-0.278211767	-0.541097856	0.420131474	0.999225654	11.52	16.9
Cd2ap	CD2-associated protein	12488	protein_coding	-0.166016337	-0.175495226	0.287506873	0.977260339	1960.51	2213.69
Cd2bp2	CD2 antigen (cytoplasmic tail) binding protein 2	70233	protein_coding	-0.104696197	-0.113343257	0.567955849	0.999225654	364.5	393.23
Cd300a	CD300A antigen	217303	protein_coding	0.154346307	0.814640085	0.570245457	0.999225654	8.48	4.71
Cd300lg	CD300 antigen like family member G	52685	protein_coding	-0.026956585	-0.158515182	0.917135714	0.999225654	9.98	10.96
Cd302	CD302 antigen	66205	protein_coding	0.02527774	0.07752775	0.937751318	0.999225654	64.87	61.3
Cd34	CD34 antigen	12490	protein_coding	-0.929274187	-3.11027247	0.004458447	0.210400686	4	33.63
Cd36	CD36 antigen	12491	protein_coding	1.930276104	5.183084628	1.99106E-08	1.36787E-05	258.74	6.73
Cd3eap	CD3E antigen, epsilon polypeptide associated protein	70333	protein_coding	-0.178841158	-0.201070896	0.408547698	0.999225654	131.1	150.77
Cd44	CD44 antigen	12505	protein_coding	1.004689674	3.311081876	0.002234191	0.156596987	53.49	5.11
Cd46	CD46 antigen, complement regulatory protein	17221	protein_coding	0.564549226	1.065787269	0.101122233	0.811028055	36.04	17.51
Cd47	CD47 antigen (Rh-related antigen, integrin-associated signal transducer)	16423	protein_coding	0.428172763	0.518727474	0.102279380	0.815977768	539.44	376.65
Cd55	CD55 antigen	13136	protein_coding	0.198146265	0.29862686	0.543336177	0.999225654	33.62	27.43
Cd59a	CD59a antigen	12509	protein_coding	0.145731098	0.217497357	0.653490111	0.999225654	790.76	680.58
Cd59b	CD59b antigen	333883	protein_coding	-0.253677443	-0.340683572	0.399882600	0.999225654	35.73	45.09
Cd63	CD63 antigen	12512	protein_coding	0.274085053	0.311952988	0.224109173	0.94643770	1740.63	1402.12
Cd63-ps	CD63 antigen, pseudogene	626721	pseudogene	-0.384136701	-0.612566518	0.249769307	0.958974344	15.33	23.51
Cd68	CD68 antigen	12514	protein_coding	0.073805033	0.615668949	0.743800339	0.999225654	10	6.56
Cd74	CD74 antigen (invariant polypeptide of major histocompatibility complex, cl	16149	protein_coding	0.628385078	1.690531839			181.33	55.84
Cd79b	CD79B antigen	15985	protein_coding	0.177903312	1.384937585	0.450721486		4.48	1.63
Cd81	CD81 antigen	12520	protein_coding	0.282518283	0.342116696	0.280628021	0.977155712	2067.41	1631.61
Cd82	CD82 antigen	12521	protein_coding	0.098797075	0.142772561	0.756603663	0.999225654	952.99	863.42
Cd83	CD83 antigen	12522	protein_coding	0.44784823	1.495289294	0.160335933	0.89602500	14.39	4.84
Cd86	CD86 antigen	12524	protein_coding	-0.272492097	-0.621289923	0.426452233	0.999225654	5.09	7.72
Cd9	CD9 antigen	12527	protein_coding	-0.497838134	-0.755394278	0.128075042	0.867190171	616.06	1040.58
Cd97	CD97 antigen	26364	protein_coding	0.141524593	0.29997726	0.681363272	0.999225654	77.18	62.27
Cd99	CD99 antigen	673094	protein_coding	-0.275714755	-0.545360752	0.424424719	0.999225654	9.88	14.72
Cd99l2	CD99 antigen-like 2	171486	protein_coding	0.118452097	0.144712941	0.656210553	0.999225654	469.69	425.18
Cxcl10	chemokine (C-X-C motif) ligand 10	15945	protein_coding	0.148805959	0.828515177	0.576912445	0.999225654	9	5.03
Cxcl12	chemokine (C-X-C motif) ligand 12	20315	protein_coding	0.179436333	0.339723661	0.602625548	0.999225654	158.25	124.94
Cxcr4	chemokine (C-X-C motif) receptor 4	12767	protein_coding	-0.145567063	-0.214303049	0.651386272	0.999225654	32.32	37.99
Cx3cl1	chemokine (C-X3-C motif) ligand 1	20312	protein_coding	0.161915433	0.228593333	0.605854561	0.999225654	495.75	423.6
Ccl25	chemokine (C-C motif) ligand 25	20300	protein_coding	0.071407669	0.088420354	0.793021603	0.999225654	56.78	53.59
Ccl27a	chemokine (C-C motif) ligand 27A	20301	protein_coding	-0.174301951	-0.243986453	0.576323428	0.999225654	90.09	107.03
Ccl28	chemokine (C-C motif) ligand 28	56838	protein_coding	0.423505219	1.415690135	0.183441303	0.918795647	17.86	6.45
Defb1	defensin beta 1	13214	protein_coding	-0.316322581	-0.502047488	0.342556832	0.998670486	5797.18	8210.46
Defb11	defensin beta 11	246081	protein_coding	0.083421381	0.346164828	0.777733086	0.999225654	13.28	10.46
Defb29	defensin beta 29	75400	protein_coding	1.061034943	3.115267151	0.001589346	0.130407149	40.79	4.82
Defb42	defensin beta 42	619548	protein_coding	0.187685138	0.732893044	0.533421459		5.62	3.21
Lcn2	lipocalin 2	16819	protein_coding	0.863692879	7.241425421	0.003369369	0.179902016	63.04	0.56
S100a1	S100 calcium binding protein A1	20193	protein_coding	-0.342638336	-0.443555458	0.236471293	0.952377311	1652.18	2247.13
S100a10	S100 calcium binding protein A10 (calpactin)	20194	protein_coding	-0.415619274	-0.52294947	0.136107714	0.871173353	302.99	434.33
S100a11	S100 calcium binding protein A11 (calgizzarin)	20195	protein_coding	-0.127188578	-0.179174858	0.684830819	0.999225654	610.39	690.88
S100a13	S100 calcium binding protein A13	20196	protein_coding	-0.130160	-0.163310988	0.639237237	0.999225654	143.08	160.85
S100a14	S100 calcium binding protein A14	66166	protein_coding	0.22392731	0.903287931	0.454318804	0.999225654	9.05	4.71
S100a16	S100 calcium binding protein A16	67860	protein_coding	-0.246904302	-0.276839839	0.249600801	0.958974344	135.03	163.55
S100a6	S100 calcium binding protein A6 (calcyclin)	20200	protein_coding	-0.241593197	-0.788462916	0.448665523	0.999225654	22.64	38.78
S100g	S100 calcium binding protein G	12309	protein_coding	-1.075535921	-1.983245919	0.001739910	0.138393229	14503.39	57344.08
S100pbp	S100P binding protein	74648	protein_coding	0.243751033	0.364717227	0.453065550	0.999225654	57.44	44.73
Cav1	caveolin 1, caveolae protein	12389	protein_coding	-0.547838912	-0.643837121	0.025898460	0.497668052	128.79	202.02
Cav2	caveolin 2	12390	protein_coding	-0.741172776	-1.073643911	0.020144761	0.446660092	213.64	449.96
Scg5	secretogranin V	20394	protein_coding	-0.220354844	-0.370344184	0.515576223	0.999225654	186.03	240.55
Clec11a	C-type lectin domain family 11, member a	20256	protein_coding	-0.234326326	-0.46648641	0.497271948	0.999225654	17.46	24.55
Clec14a	C-type lectin domain family 14, member a	66864	protein_coding	-0.09820173	-0.806165574	0.665781013	0.999225654	8.77	15.18
Clec16a	C-type lectin domain family 16, member A	74374	protein_coding	0.059839664	0.065734906	0.761715302	0.999225654	718.35	685.84
Clec1a	C-type lectin domain family 1, member a	243653	protein_coding	-0.096078349	-0.160918358	0.776808737	0.999225654	26.15	28.91
Clec2d	C-type lectin domain family 2, member d	93694	protein_coding	-0.370819723	-0.431651271	0.122546156	0.860192276	330.16	446
Clec2h	C-type lectin domain family 2, member h	94071	protein_coding	-0.225649215	-0.529397821	0.508820669	0.999225654	235.73	340
Clec3b	C-type lectin domain family 3, member b	21922	protein_coding	-0.324585592	-2.427067735	0.192398584	0.922101249	2.52	13.39
Clec9a	C-type lectin domain family 9, member a	232414	protein_coding	-0.31637432	-1.159187889	0.306155392	0.987313216	5.34	12
Tradd	TNFRSF1A-associated via death domain	71609	protein_coding	-0.162009417	-0.216486	0.588306777	0.999225654	64.99	76.17
Traf1	TNF receptor-associated factor 1	22029	protein_coding	0.474141012	1.170850	0.162979968	0.90117409	62.93	28.48
Traf2	TNF receptor-associated factor 2	22030	protein_coding	0.03177757	0.040334	0.910354968	0.999225654	208.3	202.92
Traf3	TNF receptor-associated factor 3	22031	protein_coding	-0.198455617	-0.238380	0.441367830	0.999225654	239.76	283.38
Traf3ip1	TRAF3 interacting protein 1	74019	protein_coding	-0.281078932	-0.316086	0.194415869	0.92280110	330.45	411.22
Traf3ip2	TRAF3 interacting protein 2	103213	protein_coding	-0.475398393	-0.574540	0.068562665	0.727045038	100.26	149.48
Traf4	TNF receptor associated factor 4	22032	protein_coding	-0.134092276	-0.148498	0.511813686	0.999225654	569.1	630.26
Traf5	TNF receptor-associated factor 5	22033	protein_coding	-0.026281558	-0.031826	0.920037117	0.999225654	94.16	96.99
Traf6	TNF receptor-associated factor 6	22034	protein_coding	0.224376734	0.246131	0.252577229	0.961397028	326.88	274.82
Traf7	TNF receptor-associated factor 7	224619	protein_coding	-0.193551713	-0.212204	0.321938699	0.995796675	355.09	412.7
Trafd1	TRAF type zinc finger domain containing 1	231712	protein_coding	0.200849324	0.223440	0.334830477	0.997759559	224.25	191.05
Traip	TRAF-interacting protein	22036	protein_coding	0.377492826	2.099927	0.170005104		7.34	1.59
Stat1	signal transducer and activator of transcription 1	20846	protein_coding	0.19543372	0.219045540	0.361563094	0.999225654	435.36	372.79
Stat2	signal transducer and activator of transcription 2	20847	protein_coding	0.501232328	0.580983919	0.034988941	0.556420596	167.14	111.08
Stat3	signal transducer and activator of transcription 3	20848	protein_coding	-0.191279523	-0.224436643	0.434967530	0.999225654	1062.56	1241.46
Stat5a	signal transducer and activator of transcription 5A	20850	protein_coding	0.279715766	0.335770967	0.277161329	0.975715675	265.71	211.3
Stat5b	signal transducer and activator of transcription 5B	20851	protein_coding	0.055009004	0.058109136	0.722934339	0.999225654	458.84	440.5
Stat6	signal transducer and activator of transcription 6	20852	protein_coding	0.08067932	0.090479588	0.706913731	0.999225654	755.7	709.2
Irf1	interferon regulatory factor 1	16362	protein_coding	0.166668472	0.243336962	0.603433359	0.999225654	90.34	75.8
Irf2	interferon regulatory factor 2	16363	protein_coding	-0.156778134	-0.17698409	0.475386998	0.999225654	309.76	349.33
Irf2bp1	interferon regulatory factor 2 binding protein 1	272359	protein_coding	-0.051751809	-0.058023238	0.809223139	0.999225654	311.14	324.04
Irf2bp2	interferon regulatory factor 2 binding protein 2	270110	protein_coding	-0.369760512	-0.403509772	0.053060659	0.665951737	1281.43	1694.93
Irf2bpl	interferon regulatory factor 2 binding protein-like	238330	protein_coding	-0.250108129	-0.304268205	0.343696751	0.998805722	749.84	925.5
Irf3	interferon regulatory factor 3	54131	protein_coding	0.050025209	0.055532013	0.808378894	0.999225654	333.7	321.69
Irf5	interferon regulatory factor 5	27056	protein_coding	0.302652866	1.231774459			26.02	10.97
Irf6	interferon regulatory factor 6	54139	protein_coding	0.429440846	0.588130822	0.160986901	0.897932237	328.12	218.02
Irf7	interferon regulatory factor 7	54123	protein_coding	0.299723491	0.481470351	0.370200486	0.999225654	28.91	20.75
Irf8	interferon regulatory factor 8	15900	protein_coding	-0.108383194	-0.363427552	0.731623498	0.999225654	64.58	82.82
Irf9	interferon regulatory factor 9	16391	protein_coding	0.049225256	0.064270287	0.866228938	0.999225654	164.34	158.02

S4

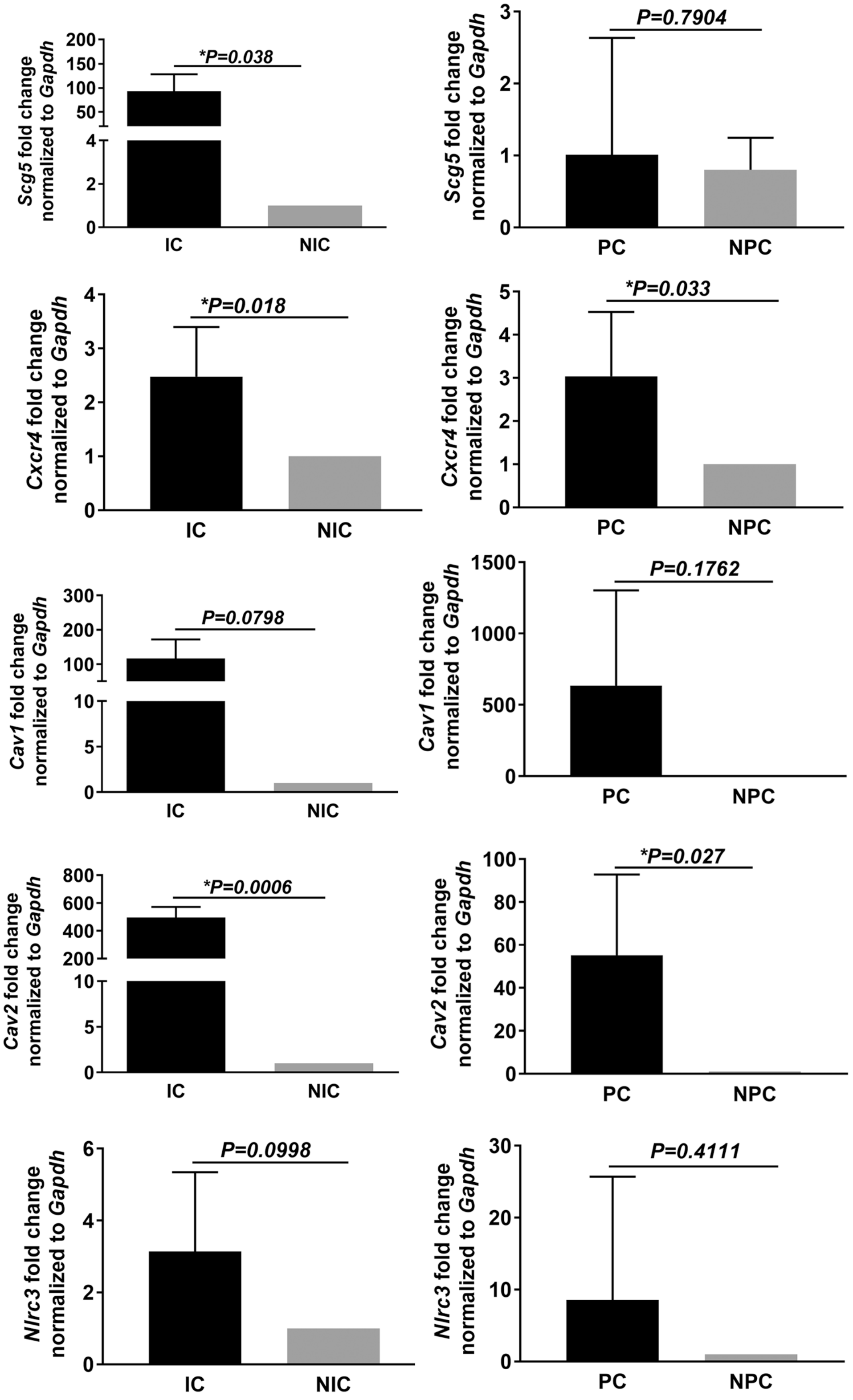

Scg5 fold change normalized to Gapdh
*P=0.038
IC
NIC
P=0.7904
PC
NPC
Cxcr4 fold change normalized to Gapdh
*P=0.018
*P=0.033
Cav1 fold change normalized to Gapdh
P=0.0798
P=0.1762
Cav2 fold change normalized to Gapdh
*P=0.0006
*P=0.027
Nlrc3 fold change normalized to Gapdh
P=0.0998
P=0.4111

S5: genes with highest expression in ICs on pilot single cell study			
Gene	Gene abbreviation	Mean read count ± std dev	Function (http://genecards.org)
Metastasis Associated Lung Adenocarcinoma Transcript 1	Malat1	13160 ± 22842	may act as transcriptional regulator for cancer metastasis, cell migration and cell cycle regulation genes
Glutathione Peroxidase 3	Gpx3	5656 ± 6808	protects cell against oxidative damage
S100 Calcium Binding Protein G	S100g	5268 ± 7032	vitamin D dependent calcium binding protein
Kallikrein 1	Klk1	4927 ± 8220	diverse functions including the release of vasoactive peptides
Calbindin 1	Calb1	2507 ± 3873	calcium binding protein
Fms Related Tyrosine Kinase 1	Ftl1	2230 ± 2331	encodes member vascular endothelial growth factor receptor (VEGFR) family.
Aldolase, Fructose-Bisphosphate B	Aldob	1905 ± 2196	catalyzes the reversible conversion of fructose-1,6-bisphosphate to glyceraldehyde 3-phosphate and dihydroxyacetone phosphate
Secreted Phosphoprotein 1	Spp1	1716 ± 1146	cytokine that upregulates expression of interferon-gamma and interleukin-12.
Ferritin Heavy Chain 1	Fth1	1703 ± 1243	encodes the heavy subunit of ferritin, the major intracellular iron storage protein in prokaryotes and eukaryotes
Myo-Inositol Oxygenase	Miox	1648 ± 2074	iron ion binding and oxidoreductase activity
Aldo-Keto Reductase Family 1 Member A1	Akr1a1	1055 ± 1270	involved in the reduction of biogenic and xenobiotic aldehydes
FXYD Domain Containing Ion Transport Regulator 2	Fxyd2	975 ± 615	encodes sodium/potassium-transporting ATPase subunit gamma
Solute Carrier Family 34 Member 1	Slc34a1	961 ± 1428	encodes a member of the type II sodium-phosphate cotransporter family
Clusterin	Clu	941± 1591	secreted chaperone
Mitochondrial ATP Synthase Beta Subunit	Atp5b	895 ± 454	encodes a subunit of mitochondrial ATP synthase
Glutathione Peroxidase 1	Gpx1	829 ± 953	Protect cell against oxidative damage
Solute Carrier Family 25 Member 5	Slc25a5	800 ± 815	mitochondrial carrier subfamily of solute carrier protein genes
Transmembrane Protein 27	Tmem27	793 ± 1341	Trafficking amino acid transporters
Integral Membrane Protein 2B	Itm2b	752 ± 487	inhibits the deposition of beta-amyloid.
Ubiquitin B	Ubb	699 ± 1051	Targets cellular proteins for degradation
